# Elements pollution and ecological risk assessment of coastal sediments along the Nile Delta

**DOI:** 10.1038/s41598-025-06801-4

**Published:** 2025-06-20

**Authors:** Mohamed A. Hassaan, Amr G. Dardeer, Ahmed El Nemr

**Affiliations:** 1 National Institute of Oceanography and Fisheries (NIOF), Kayet Bey, El-Anfoushy, Alexandria, Egypt; 2 Administration of Environmental Monitoring-Ministry of Health and Population, Alexandria, Egypt

**Keywords:** Contamination indices, Potentially toxic elements, Human risk assessment, Mediterranean Coast, Pollution load index, Sediment quality guidelines, Environmental chemistry, Environmental impact

## Abstract

The Nile Delta coastal area surface sediments were evaluated for twenty-five elements. Inductively coupled plasma-mass spectroscopy (ICP-MS method) was used to analyze the digested solutions, previously filtered using discrete 0.2 μm PTFE syringe filters according to USEPA protocols. Potentially toxic elements (PTEs) and pollution levels were estimated using several indices. Pollution guides such as the enrichment factor (EF), contamination degree (*C*_d_), geoaccumulation index (*I*_geo_), and pollution load index (PLI) are mainly determined by actually toxic elements such as Cr, Co, Cd, Hg and Pb, while Principal component analysis (PCA), concerned with the distribution of all elements in sediment to determine the sources of elements. The following is the order of the EF values: Mn < Cu < Zn < Ni < Pb < Cr. The areas under investigation showed no pollution with Cr, Cu, Mn, Ni, Pb, or Zn, as indicated by their (*I*_geo_ ≤0) values and *C*_d_ (< 1.5). Significant connections between Mn, Fe, Cr, Co, and Ni values were observed, indicating comparable origins. In the current study, the children’s Hazard Quotient (HQ) results for the dermal exposure pathway are low, but adult values are 3–4 times higher. The chronic daily intake *(CDI*_Dermal_) and carcinogenic risk (*CR*_Dermal_) through dermal absorption were also investigated.

**Supplementary Information:**

The online version contains supplementary material available at 10.1038/s41598-025-06801-4.

## Introduction

In recent years, considerable concerns have been expressed about heavy metal (HM) poisoning aquatic habitats^1–3^. Because of their resistance to natural breakdown, potentially toxic elements (PTEs) are among the most persistent contaminants in ecosystems. It turns poisonous once the degree of irreplaceability is reached. PTEs become harmful when they accumulate in soft tissues without being metabolized by the body^4,5^. In some environmental conditions, HM concentrations can rise to dangerous levels, which might harm the ecosystem^6^. Although Fe, Zn, Cu, and Mn are considered necessary metals due to their critical roles in biological processes, greater concentrations of these elements can be dangerous^7–10^. Non-essential metals like Pb and Cd can lead to intoxication, tissue and cellular damage, decreased fertility, organ dysfunction, and even cell death when they bioaccumulate in tissues. Even at extremely low doses, these metals are frequently potent toxins^11–14^. Lead and cadmium are included in the hazardous metal criteria of the European Union, and nickel and chromium have been classified as harmful metals by the US Food and Drug Administration^15,16^. Metals are adsorbed and build up on bottom sediments because they are not very soluble in water^4^. Metals that have settled in sediments in the aquatic environment may re-suspend and further contaminate the water since sediments are both a source and a sink of metals^17^. Because of this feature, the sediments become an enduring record of the anthropogenic pollutants that have been added^9,18,19^. Thus, spatial surveys of metal concentrations in sediments and comparison with uncontaminated baselines are essential to understanding the mechanisms behind the accumulation and geochemical distribution of PTEs in aquatic systems and to provide critical information for evaluating risks to human health^20,21^.

The ecological dangers associated with PTEs have been evaluated using various techniques thus far. Most of them, meanwhile, like the Geoaccumulation index (*I*_geo_) approach and Enrichment factor (*EF*), are only suitable for the ecological assessment of a single contaminant. A lot of different kinds of PTEs usually accumulate at the same time and cause combined pollution. SQG was developed, nonetheless, to assess the potential effects of pollutant mixtures in sediments^22^.

Regretfully, most of Egypt’s Mediterranean coastal zones get substantial pollution due to numerous human activities^23–27^. Eight coastal governorates around Egypt’s Mediterranean coast from west to east, including Matruh, Alexandria, Behaira, Kafr El-Sheikh, Damietta, Daqahliya, Port Said, and North Sinai. The pollutant load that reaches the coastal waterways is increased by the metro area’s high population and the numerous agricultural regions surrounding it. Either the drainage canals that discharged directly into the sea, like “El-Tabia and El-Ummum,” the irrigation canals of Mahmudiya and Nubariya, the Rosetta branch of the River Nile, or the “lakes” of coastal lagoons, like Maryut, Idku, Burullus, and Manzala, were the direct sources of these pollutants. Despite appropriate mitigation and protection measures, significant portions of the Nile Delta have been negatively harmed by coastal erosion. However, most coastal lagoons, or “lakes,” are endangered because of excessive sewage from residences, companies, and farms^23,27,28^.

Egypt’s coastal Nile Delta region is a sensitive and dynamic ecosystem impacted by various human activities, including ports, industrial areas, aquaculture, agriculture, tourism, and other activities. In addition, there are other environmental issues along the Nile Delta’s shoreline, including pollution, erosion, decreased productivity, and coastal deterioration^3,4^. In the northern portion of the Nile Delta, runoff from various urban, industrial, and agricultural sources is collected by a sophisticated drainage system. The drainage network discharges partially treated urban waste into the branches of the Nile and coastal lagoons. These wastes eventually enter the Mediterranean by direct drains like the Kitchener and El-Tabia drains or the coastal lagoon outlets.

Because of the apparent effects of land-based activities on the coastal ecosystem of the Nile Delta, many researchers have become aware of the pollution of coastal sediments by trace metals^27,29–31^. These specialists claim that trace metal contamination exists in certain drains, coastal lagoons, and the water and sediments of the coastal zone of the Nile Delta. Abu Qir Bay to the west of the Nile Delta and the coastal lagoons of the Delta are hotspots for metal pollution^4^. Last but not least is the Bardawil Lagoon, a naturally occurring depression on the Sinai Peninsula’s north shore. Zaranik’s natural eastern entrance, which is now occasionally blocked by silting, and two artificial tidal inlets (270 and 300 m wide and 4–7 m deep), which are continuously dredged, are the three inlets that enable seawater to enter the lake^26^. Due mainly to its European exports, the Bardawil Lagoon enjoys a strong international reputation and substantial economic effect from its high-quality fish production^26^. These industries, which include fishing, industrial, agricultural, and residential water and sewage systems, have been selected and exposed to many point sources of contamination.

The regions affected by PTEs are often restricted to highly cultivated areas and on the outskirts of large cities or industrialized regions^30–32^. Introducing PTEs into the ecosystem can affect marine organisms’ ability to photosynthesize, reproductive cycles, cell growth and regeneration, and sediment nutrient cycling^33^. Examples of PTEs that are helpful markers of pollution caused by human activities are Pb and Cd^35,36^. Furthermore, Cadmium (Cd) may be present in some phosphate-containing fertilizers that might be a significant source of dietary Cd absorption for humans. Additionally, during wastewater treatment, sewage sludge contains substantial levels of Cd^37^. The term “leachable metal fraction” refers to the proportion of metals that are anthropogenically present in sediment particles. Diluted acids have often removed leachable components^38–41^.

This study’s aims are as follows: (i) evaluate the distribution and concentrations of twenty-five elements in sediments collected from the Nile Delta coastal area; (ii) evaluate the degree of HM contamination and enrichment in the sediment using the Pollution Load Index (PLI), Enrichment Factor (*EF*), Contamination degree (*C*_d_), Geoaccumulation Factor (*I*_geo_), and the sediment quality guidelines (SQGs) to determine the ecological risk of these potentially toxic metals like Cr, Pb, Cu, Ni, Cd, Hg, and Mn (iii) conduct multivariate statistical analysis using methods like principal component analysis (PCA) and Pearson’s correlation matrix; (iv) evaluate the possible health risk of cancer by calculating the amounts of metals (Cd, Fe, Cu, Mn, Zn and Pb) based on dermal exposure pathways. The current study is the first to report the monitoring and measuring of 25 elements in the studied area collected sediments.

## Materials and methods

### Study area

**Fig. 1:**
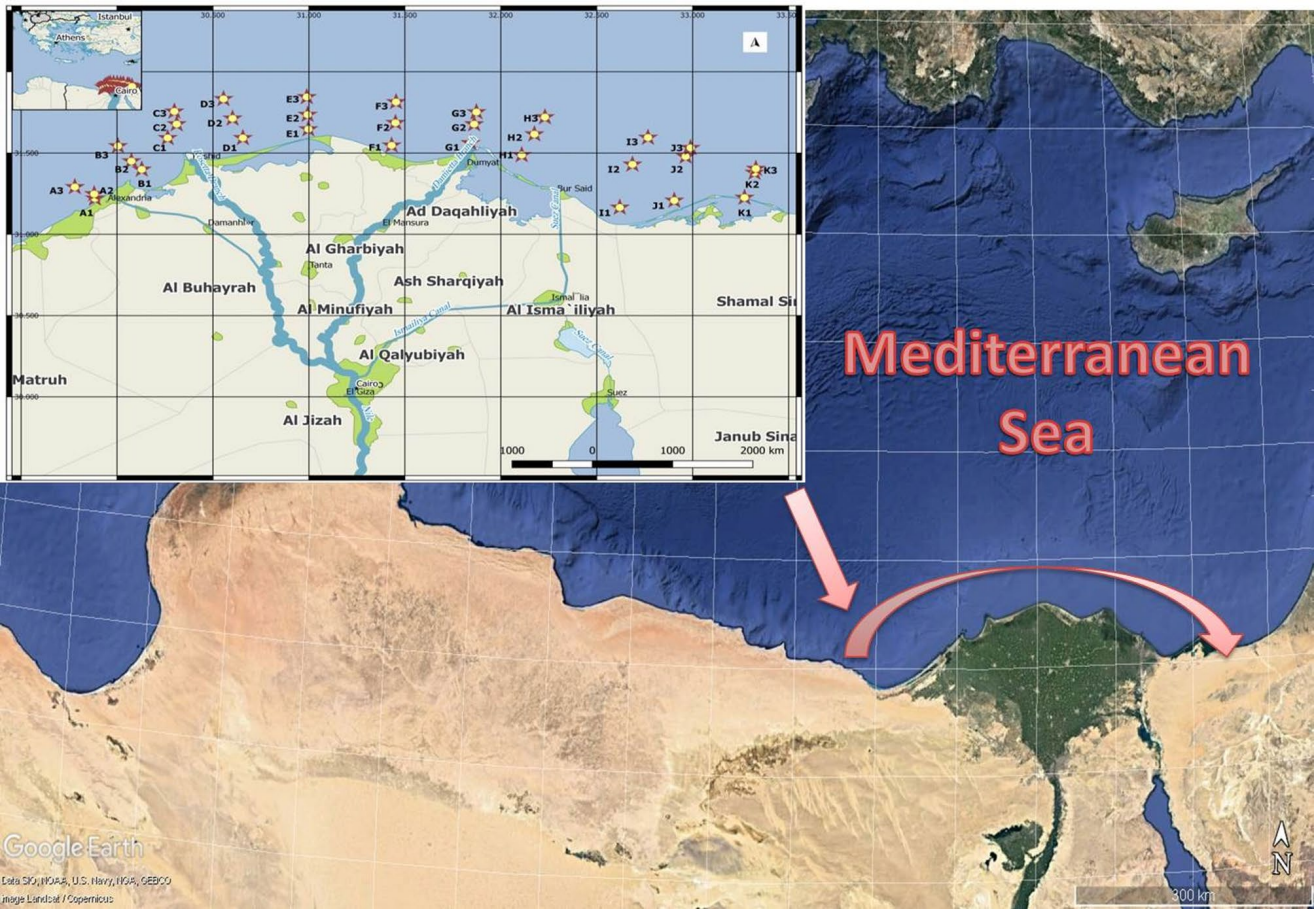
The study area is located between 31,404′ N–33,329′ E and 31,221′ N–29,885′ E (Software QGIS 3.18; https://www.filehorse.com/download-qgis/61739/).

The research area, as seen in Fig. 1, covers the coastal region of the Nile Delta between 31,404′N–33,329′E and 31,221′N–29,885′E. Four coastal lagoons in this region—Edku, Burullus, Manzala, and Bardaweel—were flooded by runoff from the delta drainage system^31^. Sandy and silty shorelines with various lateral formations sculpted by the historical discharge places of the old Nile branches make up the Nile Delta coastline, which stretches from Alexandria to Bardaweel Lagoon. Along the coastline, the Rosetta and Damietta promontories are notable landmarks. The beaches surround four shallow lakes (Mariut, Edku, Burullus, and El Manzalah) and coastal flats that give way to coastal dunes. Due to its low land elevation and local ground subsidence, this area is especially susceptible to sea level rise. According to geology, The Delta comprises feldspars, loose quartz sand, and significant amounts of HM and shell pieces^31^. The region is further impacted by wastewater drainage from the El-Tapia pumping station, freshwater inflows from Lake Edku and Lake Burullus, and the Rosetta and Damietta branches of the Nile^31^.

### Samples collection and Preparation

The NIOF research vessel Yarmouk used a Van Veen grab sampler (Aquatic BioTechnology S.L., Spain) to gather all of the samples from the eleven northern Mediterranean sections between Alexandria and Port Said. Surface sediment samples were obtained from 31 locations with depths ranging from 10 to 50 m (Fig. 1, Table S1). Each section has three places: 1, 2, and 3. Conversely, Section A is divided into two locations: Section D, which is located in this area and has gas pipes, and Section A, which is located on a rocky site. Eleven sections of sediments represented the three sectors of the coastal region of the Nile Delta: the eastern sector (G, H), the middle sector (D, E, F), and the western sector (A, B, C). The last three sections (I, J, and K) encompassed Bardaweel Lake and the entrance to the Suez Canal along the eastern Egyptian border. Within a week after sampling, the samples were taken to the lab in amber glass containers and stored in a refrigerator with ice packs. At each of the chosen sampling sites, three duplicates were collected. While additional testing was being done, the samples were kept at 4 °C in a guarded icebox on the way to the NIOF Laboratory.

### PTEs analysis

The digested solutions were filtered using discrete 0.2 μm PTFE syringe filters (DISMIC-25HP, Advantec, Tokyo, Japan). The metal levels of these filtrates were measured by ICP-MS (inductively coupled plasma-mass spectroscopy; iCAP, Thermo, Germany) using the protocols described in USEPA^42^. In short, 0.2 g of the sediment samples were digested in a 1:4:1 ratio using 1 ml H_2_O_2_ (30%, Suprapur), 4 ml HNO_3_ (65%, Suprapur), and 1 ml HCL (36%, Suprapur). In a microwave digester, samples were broken down for ninety minutes at 195 °C. After being digested, the samples were filtered, and 10 milliliters of Milli Q water were added^42^. ICP-MS was used to determine the metal concentrations in the extracted samples. The investigations employed certified reference materials from Merck in Germany. The metal recovery stayed within the allowed range of 10–1000 parts per billion. The final concentration was obtained by diluting the solution with 0.1 mM HNO_3_ and using the final dilution factors. Thermo Fisher Scientific’s Qtegra software determined the average and relative standard deviation (USA).

A Quality Control and Quality Assurance (QC/QA) program was created and implemented to guarantee trustworthy results. As part of the QC/QA procedure, all measurements had to be done in triplicate, and the results had to be reported as mean value RSD. The data was considered acceptable when the three duplicate samples’ percentage variation from the RSD was less than 5%. Analytical results below the detection limit (BDL) were controlled by EPA guidelines^42^. To ensure analytical reliability, the identical sample analysis technique was used to analyze the certified reference materials SRM 2702 (NIST, USA). All element recoveries fell between 97.1% and 100.9% of their original total (Table S2)^42^.

### Pollution quantification

Several criteria were used to assess the ecological danger and sediment pollution. Numerical sediment quality recommendations (SQGs) are a standard interpretive tool to assess certain substances’ ecological risks or biological significance^43,44^. Determining if the quantities of PTEs in the sediments under examination threaten aquatic life is essential. For this evaluation, two sets of Sediment Quality Guidelines (SQGs) are used: Probable Effect Concentrations (PECs) that include the Effect Range Medium (ERM), representing concentrations above which adverse effects on sediment-dwelling organisms are frequently observed; the Probable Effect Level (PEL), a benchmark beyond which toxic effects are highly probable; and logistic regression of T50, which identifies the concentration at which there is a 50% probability of observing adverse effects. These metrics help determine contaminant levels likely to cause detrimental effects. Additionally, Threshold Effect Concentrations (TECs) include the Effect Range Low (ERL), identifying concentrations below which adverse effects are rarely observed; the Threshold Effect Level (TEL), a value below which toxic effects are unlikely; and logistic regression of T20, indicating concentrations where there is a 20% probability of adverse effects are used to establish contaminant levels unlikely to harm sediment-dwelling organisms^45–48^.

The 12 elements for calculating pollution indices, such as the EF, *C*d, PLI and *I*_geo_, are selected based on their ecological significance, potential toxicity, and geochemical relevance. These elements are commonly associated with both natural processes and anthropogenic activities, making them reliable indicators of environmental pollution. Al and Fe are included as reference elements due to their natural abundance and stability, which provide a baseline for enrichment calculations. The chosen elements align with established methodologies and international guidelines for monitoring sediment contamination, ensuring meaningful comparisons with previous studies and reflecting the pollution levels in the study area accurately.

The *I*_geo_ for the metal under investigation was calculated using Eq. (1).

1 $$\:{I}_{\text{g}\text{e}\text{o}\:}=\:{\text{l}\text{o}\text{g}}_{2}\left(\:\frac{{C}_{\text{n}}}{1.5{B}_{\text{n}}}\:\right).$$

Where *C*_n_ is the measured HM content and *B*_n_ is the baseline shale value^48^. There are seven classes in the *I*_geo_: Practically unpolluted sediment was classified as class 0 (*I*_geo_ ≤ 0), polluted to moderately polluted sediment was classified as class 1 (0 < *I*_geo_ < 1), moderately to heavily polluted sediment was classified as class 2 (1 < *I*_geo_ < 2), moderately to heavily polluted sediment was classified as class 3 (2 < *I*_geo_ < 3), heavily polluted sediment was classified as class 4 (3 < *I*_geo_ < 4), heavily polluted sediment was classified as class 5 (4 < *I*_geo_ < 5), extremely polluted sediment was classified as class 6 (*I*_geo_ > 5)^48^.

The enrichment factor (EF) values for the metals under examination around the shale average^49^ were interpreted by Birth’s proposal^50^ in Eq. (2).

2 $$\:EF=\frac{{\left(\frac{\text{X}}{\text{A}\text{l}}\right)}_{\text{s}\text{e}\text{d}\text{i}\text{m}\text{e}\text{n}\text{t}}}{{\left(\frac{\text{X}}{\text{A}\text{l}}\right)}_{\text{s}\text{h}\text{a}\text{l}\text{e}}}.$$

where x/Al is the ratio of the element to Al.

The *PLI* is a straightforward method of illustrating the extent of sediment damage from metal accumulation. *PLI* was determined using Eq. (3).

3 $$\:PLI\:={({CF}_{1}^{\text{i}}\times\:{CF}_{2}^{\text{i}}\times\:{CF}_{3}^{\text{i}}\dots\:\dots\:{CF}_{\text{n}}^{\text{i}})}^{1/\text{n}}.$$

where *CF* stands for the amount of contamination and n for the number of metals. Degraded sediments are indicated by *PLI* > 1, baseline values are shown by *PLI* = 1, and ideal conditions are shown by PLI < 1^51^. Equation (4) is used to calculate the *C*_d_, which quantifies the total effects of all metals and evaluates the relative contamination of each metal individually^52^:

4 $$\:{C}_{\text{d}}=\sum\:_{\text{i}=1}^{\text{n}}{C}_{\text{f}\text{i}}.$$

where *C*_fi_ was obtained using Eq. (5):

5 $$\:{C}_{\text{f}\text{i}}=\left(\frac{{\text{C}}_{\text{A}\text{i}}}{{\text{C}}_{\text{N}\text{i}}}\right)-1.$$

While *C*_Ni_ is the highest allowable *i* value for the metal, *C*_Ai_ is the *i* value attained. N stands for normative value, while the *C*_fi_ represents the metal’s contamination factor. From the resultant *C*_d_ values, three classes are produced: low (*C*_d_ < 1), medium (*C*_d_ = 1–3), and high (*C*_d_ > 3).

High concentrations of PTEs combined with prolonged exposure to tainted water provide a serious health danger^53^. The three major ways individuals can be exposed to PTEs are ingestion, inhalation, and skin absorption. Skin contact with polluted salt water poses a greater risk to the health of those engaged in water-related activities than does ingestion or inhalation^53–55^. In the current investigation, the health risks accompanying HM exposure for women, men and children were assessed using carcinogenic and non-carcinogenic risk factors^8,56^. Equations (6–9) were used to calculate the cutaneous risk for each group [56; 8]:

6 $$\:{CDI}_{\text{d}\text{e}\text{r}\text{m}\text{a}\text{l}}\:=\:\frac{\text{C}\:\times\:\:\text{S}\text{A}\:\times\:\text{S}\text{L}\:\times\:\:\text{A}\text{B}\text{F}\:\times\:\:\text{E}\text{D}\:\times\:\:\text{E}\text{F}\:}{\text{B}\text{W}\:\times\:\:\text{A}\text{T}}\:.$$

AS is the exposed skin area, SL is the skin adhesion factor, ABF is the skin adsorption factor, EF is the exposure frequency, ED is the exposure duration, BW is the average body weight, and AT is the average time. *CDI*_Dermal_ is the chronic daily intake. C is the concentration of PTEs in the sediment.

7 $$\:{HQ}_{\text{d}\text{e}\text{r}\text{m}\text{a}\text{l}\:}=\frac{{CDI}_{\text{d}\text{e}\text{r}\text{m}\text{a}\text{l}}}{{RfD}_{\text{d}\text{e}\text{r}\text{m}\text{a}\text{l}}}\:.$$

where HQ hazard quotient, RFD reference dose

8 $$\:\text{H}\text{I}\:=\:\sum\:_{\text{i}=1}^{\text{n}}\:{\text{H}\text{Q}}_{\text{i}}.$$

Where the HI hazard index

9 $$\:{CR}_{\text{d}\text{e}\text{r}\text{m}\text{a}\text{l}\:}=\:{CDI}_{\text{d}\text{e}\text{r}\text{m}\text{a}\text{l}\:}\times\:\text{C}\text{S}\text{F}.$$

CSF is the cancer slope factor, and *CR*_Dermal_ is the carcinogenic risk resulting from the dermal absorption of PTEs in sediment. Table S3 contains the reference dose (dermal) and slope factor for the various PTEs.

### Statistical analysis and identification of sources of PTEs

SPSS Version 19 was used for correlation and multivariate analyses in the study’s statistical analysis.

## Results and discussion

### Distribution of different elements in sediment

**Fig. 2:**
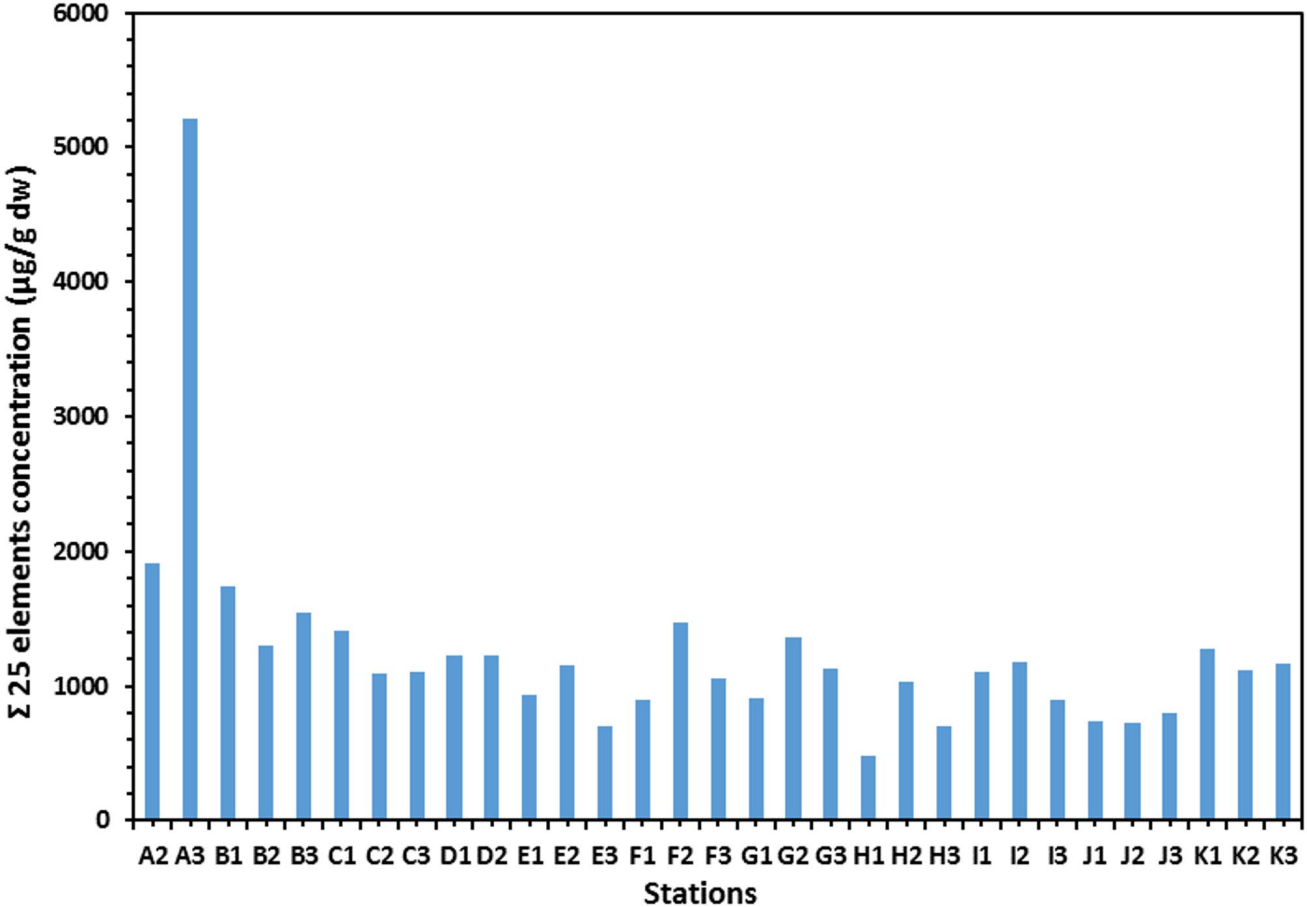
Total twenty-five elements concentration (µg/g dw) in sediment samples at different locations.

**Fig. 3:**
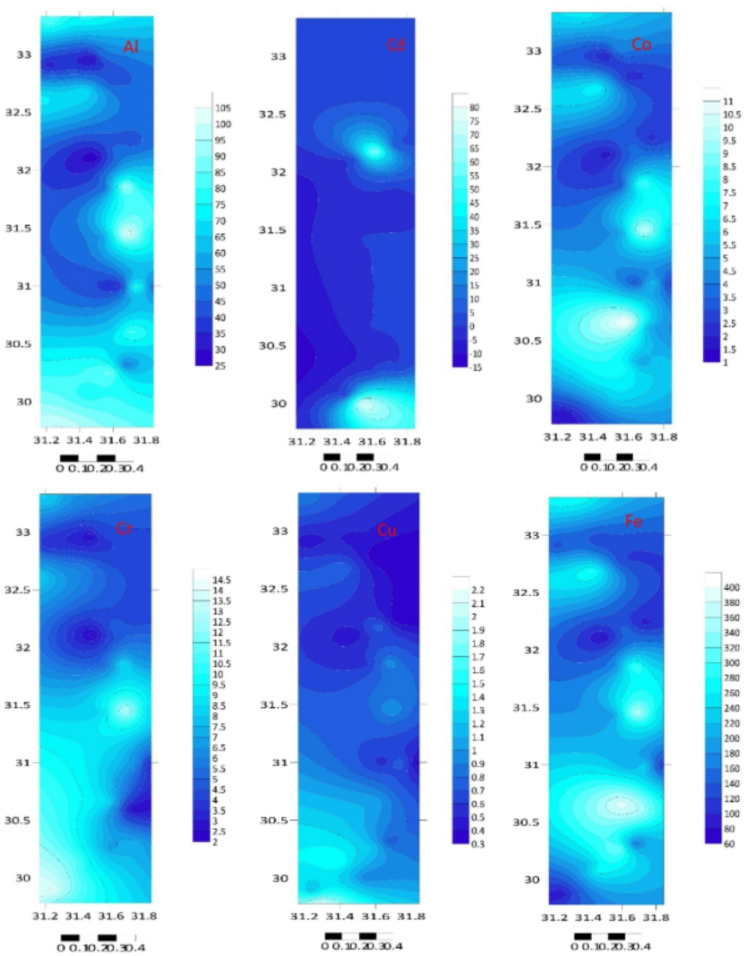

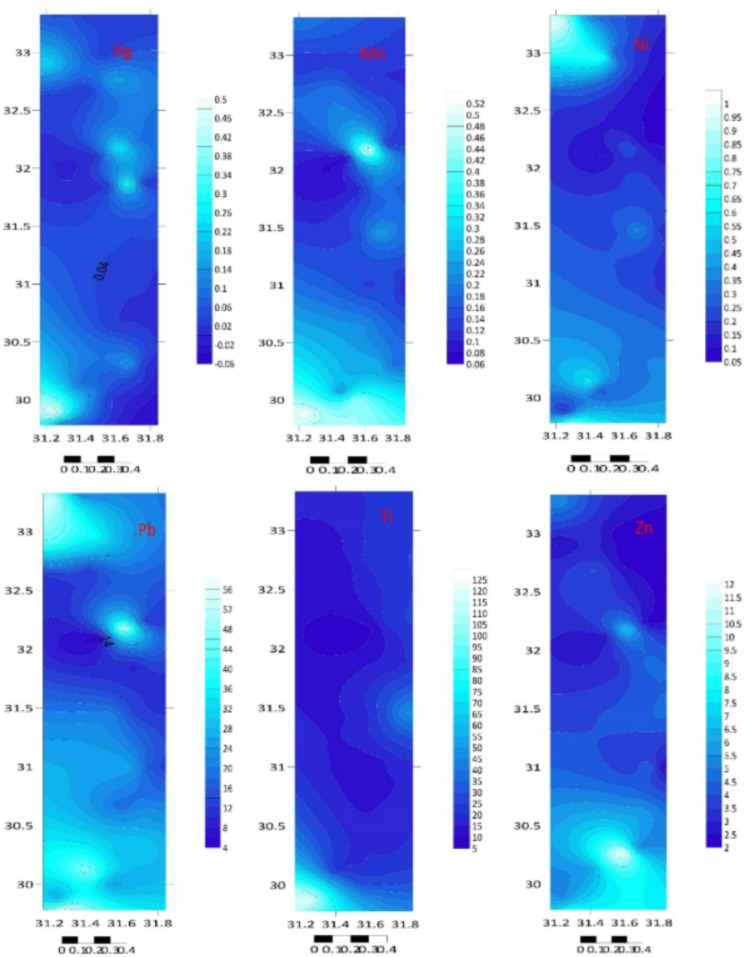

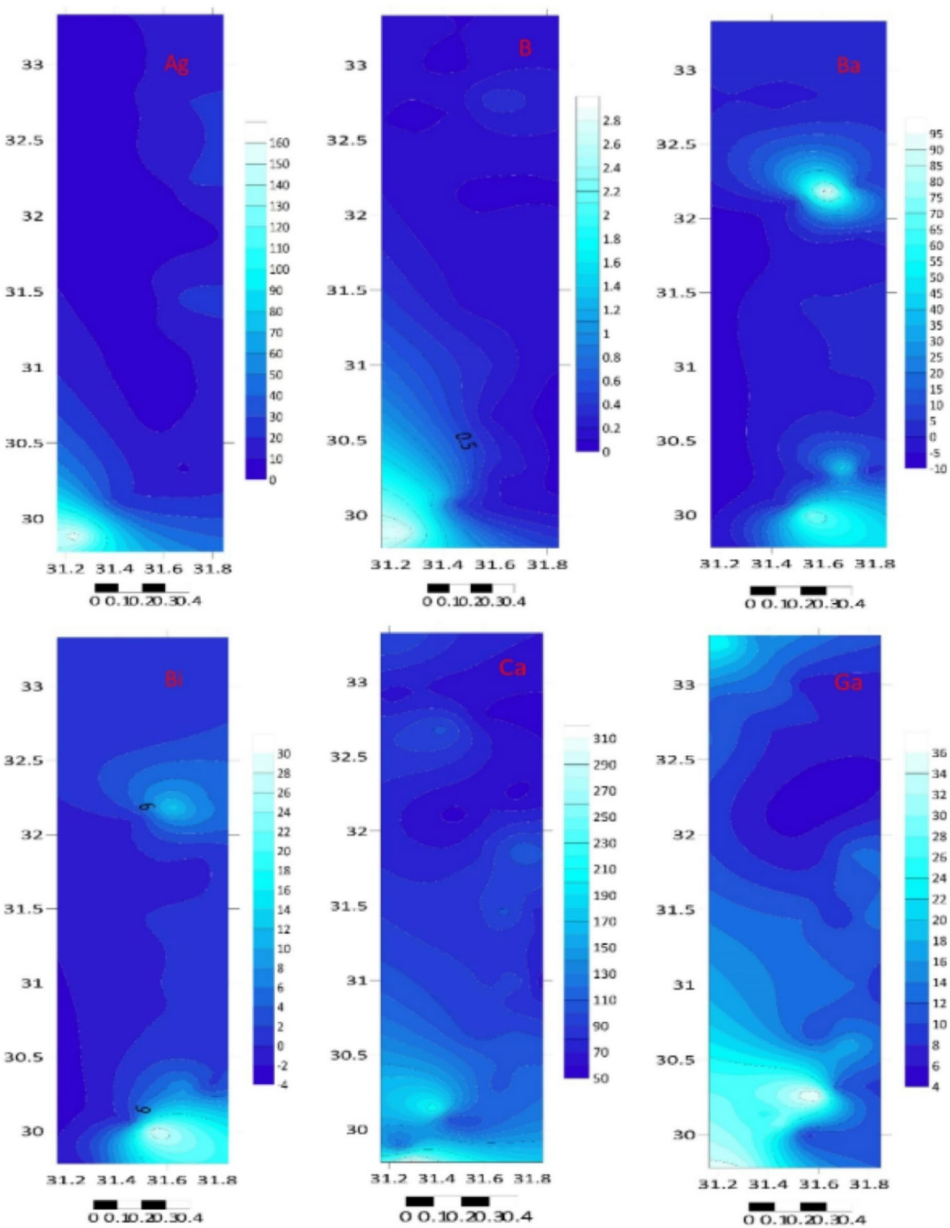

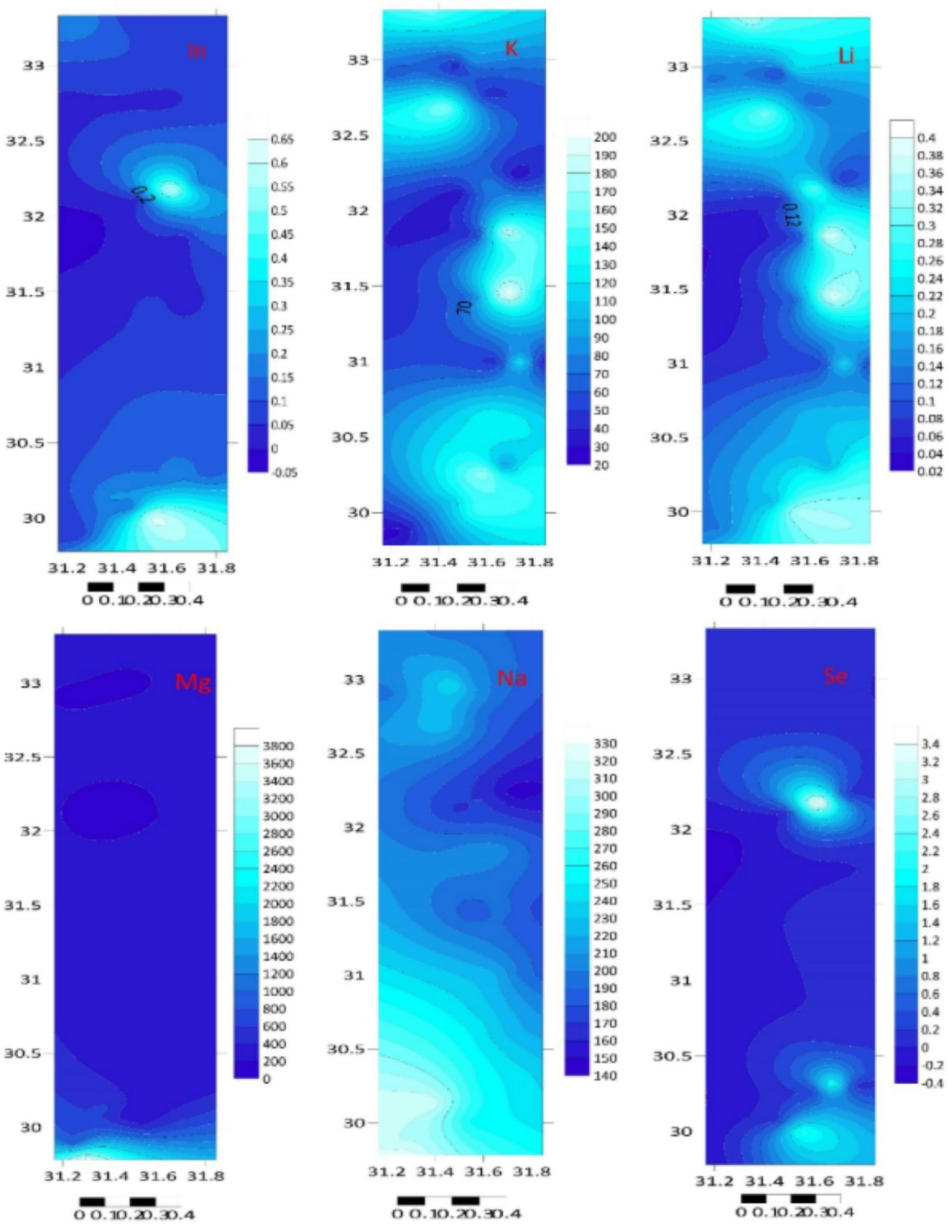
The studied metal distribution maps of the Egyptian southeastern Mediterranean sediments (Surfer 15.3 software: https://surfer.software.informer.com/15.3/).

The total element concentrations in sediment samples of the Mediterranean coast are given in Table 1; Figs. 2 and 3. Metal contents were ranging (µg/g) over subsequent averages: B: 0.086–2.844, Li: 0.066–0.409, Na: 44.550–326.769, Al: 22.810–106.953, Mg: 99.781–3969.03, K: 23.088–209.84, Ti: 6.637–123.838, Ca: 51.191–323.534, Cr: 2.217–14.186, Mn: 0.075–0.535, Co: 1.58–11.561, Fe: 74.963–395.854, Ni: 0.085–0.975, Zn: 2.005–12.457, Cu: 0.286–2.225, Ga: 4.744–38.773, Sr: 0.46–4.771, Se: 0.016–3.857, Ag: 3.326–159.387, In: 0.027–0.653, Cd: 0.136–79.701, Ba: 0.194–106.112, Pb: 5.137–58.449, Hg: 0.008–0.502 and Bi: 0.103–29.594 µg/g. The standard deviation was applied to illustrate the degree of spreading distribution of many components^57^. Since the metal concentration varies from location to location, the variability of deposition conditions, hydrodynamic processes, and terrestrial inputs may be held accountable^58^.

**Table 1:** The mean concentrations of the 25 elements detected (µg/g dry weight) in marine sediments of the studied locations.

	Li	B	Na	Mg	Al	K	Ca	Ti	Cr	Mn	Fe	Co	Ni
A2	0.14	2.84	309.42	903.60	95.53	27.50	117.26	123.84	14.19	0.52	85.20	1.58	0.16
A3	0.20	2.01	326.77	3969.03	100.85	46.05	323.53	93.72	11.06	0.45	129.20	1.71	0.83
B1	0.20	1.37	317.54	642.74	79.99	112.78	217.37	21.48	11.02	0.29	216.85	6.51	0.61
B2	0.19	0.49	290.26	296.24	74.55	108.97	100.38	30.37	9.81	0.22	273.30	6.14	0.29
B3	0.35	0.36	257.81	404.03	81.46	143.13	108.40	30.14	9.39	0.44	234.04	6.35	0.31
C1	0.25	0.21	255.91	311.39	87.99	171.19	113.47	20.40	6.73	0.25	323.00	7.15	0.33
C2	0.18	0.27	249.43	299.60	48.19	85.07	104.65	18.78	7.24	0.26	150.02	4.66	0.30
C3	0.27	0.13	245.51	245.37	61.67	131.77	118.27	20.06	7.43	0.19	211.35	6.18	0.37
D1	0.19	0.17	242.72	226.43	62.49	125.75	91.35	13.56	8.41	0.22	395.85	11.56	0.33
D2	0.20	0.13	225.56	234.71	83.00	128.55	107.59	19.14	2.53	0.19	356.33	5.16	0.33
E1	0.08	0.27	222.93	286.22	33.24	40.48	88.08	10.71	7.55	0.13	193.71	3.02	0.23
E2	0.23	0.12	200.30	322.38	83.44	126.82	97.93	18.84	6.72	0.17	233.41	5.98	0.24
E3	0.08	0.11	187.08	234.49	43.54	23.09	60.42	15.96	3.09	0.09	92.65	2.21	0.14
F1	0.12	0.12	182.98	238.29	57.20	75.39	82.19	16.43	8.49	0.14	188.24	4.69	0.23
F2	0.40	0.19	194.75	380.54	106.95	209.84	104.86	29.07	13.39	0.24	372.31	10.54	0.38
F3	0.29	0.12	176.12	244.70	74.95	122.77	76.31	47.06	8.82	0.19	246.25	6.32	0.18
G1	0.09	0.10	205.91	295.52	49.14	49.42	75.82	14.97	5.21	0.11	179.19	3.16	0.19
G2	0.41	0.15	202.82	398.65	98.26	187.21	95.53	16.83	8.78	0.21	311.88	8.45	0.25
G3	0.30	0.14	192.72	301.08	72.03	148.17	108.90	17.64	6.60	0.18	237.78	6.56	0.20
H1	0.07	0.09	167.16	99.78	25.17	32.95	54.88	6.64	2.22	0.08	74.96	1.69	0.09
H2	0.32	0.09	170.63	216.27	47.64	83.40	70.74	10.09	5.02	0.54	151.69	3.81	0.26
H3	0.07	0.09	143.72	245.89	42.43	28.95	56.72	23.94	4.22	0.13	90.87	2.26	0.09
I1	0.24	0.10	209.90	276.70	73.22	131.31	88.77	15.25	8.16	0.18	252.95	6.45	0.23
I2	0.32	0.10	228.10	261.01	70.50	168.32	105.31	11.57	5.91	0.20	291.16	7.13	0.26
I3	0.12	0.38	192.13	351.44	41.77	43.78	59.00	22.49	4.23	0.12	122.75	2.18	0.19
J1	0.13	0.12	194.58	170.27	31.98	62.93	65.24	10.87	3.36	0.12	128.29	3.08	0.58
J2	0.09	0.09	241.15	158.51	27.34	39.16	63.80	15.98	2.51	0.13	114.84	1.70	0.70
J3	0.23	0.12	210.97	174.19	47.30	86.13	67.37	19.56	4.18	0.14	137.21	4.19	0.30
K1	0.26	0.13	209.27	347.14	83.03	137.90	98.67	18.79	8.04	0.19	270.85	6.30	0.98
K2	0.36	0.11	198.39	245.35	73.19	158.44	89.18	14.64	6.43	0.18	279.15	6.89	0.37
K3	0.31	0.10	196.77	309.61	81.98	151.07	84.74	18.31	6.68	0.18	258.02	6.43	0.34
Mean	0.22	0.35	220.94	422.30	65.81	102.85	99.89	24.74	7.01	0.22	213.01	5.16	0.33
SD	0.10	0.61	44.55	674.40	22.81	53.23	51.19	23.99	3.03	0.12	88.94	2.56	0.21
Max	0.41	2.84	326.77	3969.03	106.95	209.84	323.53	123.84	14.19	0.54	395.85	11.56	0.98
Min	0.07	0.09	143.72	99.78	25.17	23.09	54.88	6.64	2.22	0.08	74.96	1.58	0.09

	Cu	Zn	Ga	Se	Sr	Ag	Cd	In	Ba	Hg	Pb	Bi	
A2	1.23	5.37	32.31	0.02	1.18	159.39	0.14	0.05	0.48	0.50	25.19	0.26	
A3	2.23	8.41	34.31	0.04	0.89	106.48	0.18	0.27	0.31	0.05	58.45	0.10	
B1	1.52	5.80	24.44	0.07	1.11	26.17	0.20	0.23	0.46	0.08	46.54	0.21	
B2	1.25	6.92	30.95	0.07	0.98	28.72	0.52	0.13	0.49	0.14	41.88	0.12	
B3	0.91	7.98	8.58	2.17	1.15	34.85	79.70	0.65	75.17	0.03	24.17	29.59	
C1	1.20	12.46	38.77	0.19	0.97	18.47	0.75	0.15	2.88	0.08	27.87	6.19	
C2	0.72	5.37	10.15	2.30	4.77	8.42	3.64	0.20	52.00	0.15	30.47	8.46	
C3	0.88	5.60	10.44	0.08	0.87	13.68	0.24	0.10	0.57	0.02	29.06	1.11	
D1	1.08	5.53	9.84	0.09	0.92	6.06	0.18	0.14	0.78	0.02	18.33	0.56	
D2	0.86	5.86	17.90	0.08	0.67	14.48	0.30	0.15	0.40	0.03	23.63	0.34	
E1	0.52	3.50	12.53	0.03	1.09	3.59	0.44	0.08	0.30	0.02	26.20	0.33	
E2	0.82	4.77	12.34	0.07	0.98	11.19	4.91	0.06	0.24	0.03	16.51	0.23	
E3	0.29	2.01	7.86	0.02	0.61	16.51	0.47	0.06	0.26	0.01	13.12	0.20	
F1	0.75	4.16	12.03	0.05	0.94	6.15	0.22	0.06	0.83	0.05	21.34	0.20	
F2	0.99	4.06	7.74	0.11	0.84	23.43	0.18	0.03	0.26	0.04	12.75	0.24	
F3	0.79	4.78	13.48	0.10	0.85	22.16	0.19	0.07	1.44	0.05	11.90	0.21	
G1	0.58	3.33	6.65	0.03	0.81	6.30	0.15	0.04	0.22	0.02	11.20	0.14	
G2	0.99	4.31	9.64	0.10	0.71	7.21	0.24	0.03	0.19	0.32	8.93	0.16	
G3	0.78	4.76	12.87	0.08	0.93	6.40	0.30	0.14	0.29	0.03	13.60	0.14	
H1	0.42	2.59	4.74	0.02	1.69	3.33	0.23	0.04	0.30	0.01	5.14	0.15	
H2	0.78	7.17	4.88	3.86	1.11	7.76	67.60	0.64	106.11	0.26	55.80	13.80	
H3	0.31	2.27	5.09	0.18	0.65	21.83	6.02	0.18	11.27	0.10	12.79	6.52	
I1	0.72	3.33	12.30	0.07	0.73	6.76	1.58	0.03	0.53	0.03	10.79	0.94	
I2	0.78	4.13	7.77	0.09	0.61	4.20	1.07	0.05	0.46	0.02	13.59	0.34	
I3	0.32	2.19	8.11	0.03	0.48	19.61	0.60	0.03	0.24	0.16	22.75	0.32	
J1	0.43	2.44	11.07	0.04	0.69	4.48	0.84	0.06	0.25	0.23	43.61	0.26	
J2	0.47	3.04	11.50	0.03	0.55	6.42	1.16	0.08	0.21	0.05	36.54	0.30	
J3	0.51	2.29	6.47	0.06	0.53	10.59	0.84	0.10	1.01	0.03	29.27	0.33	
K1	0.87	5.39	23.55	0.12	0.46	8.39	3.52	0.19	2.18	0.06	53.79	1.63	
K2	0.71	4.05	13.81	0.14	0.51	5.58	1.33	0.13	3.86	0.06	19.47	1.05	
K3	0.73	3.78	16.80	0.08	0.50	9.59	0.32	0.06	0.22	0.03	22.43	0.32	
Mean	0.82	4.76	14.16	0.34	0.96	20.26	5.74	0.14	8.52	0.09	25.39	2.41	
SD	0.39	2.21	9.13	0.85	0.76	31.92	18.25	0.15	24.17	0.11	14.48	5.90	
Max	2.23	12.46	38.77	3.86	4.77	159.39	79.70	0.65	106.11	0.50	58.45	29.59	
Min	0.29	2.01	4.74	0.02	0.46	3.33	0.14	0.03	0.19	0.01	5.14	0.10	

### Assessment of sediment pollution indices

**Fig. 4:**
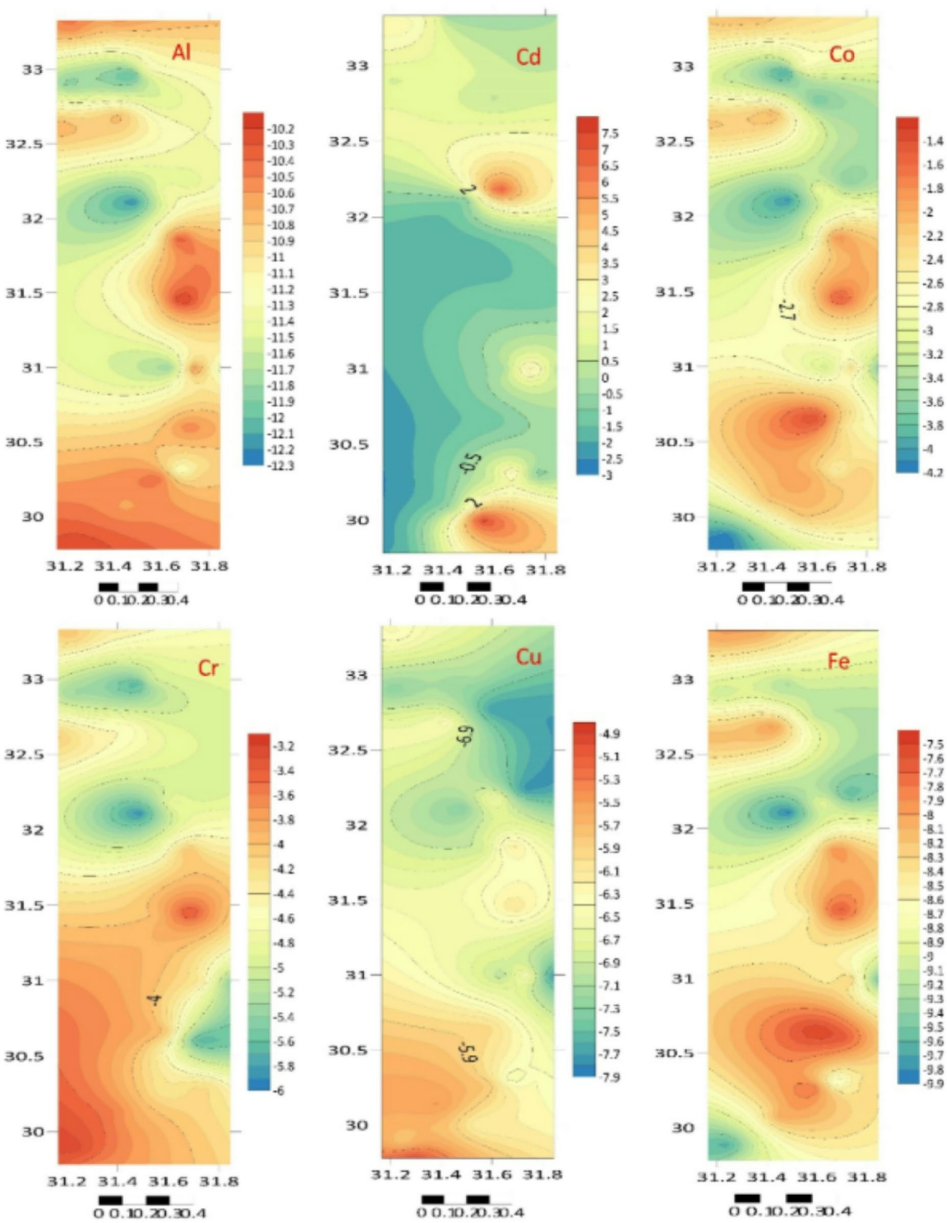

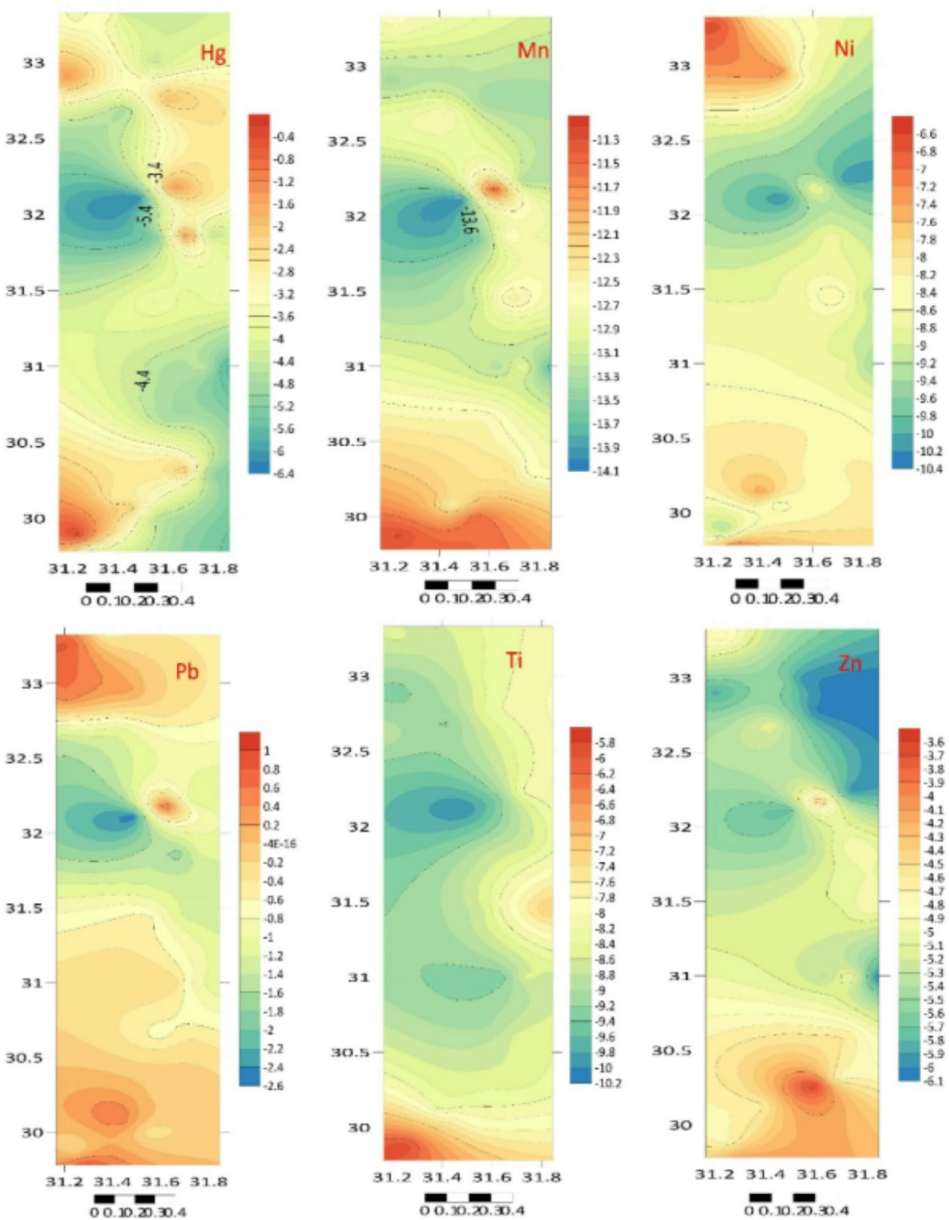
The distributions of *I*_geo_ in the Nile Delta coastal area sediments (Surfer 15.3 software: https://surfer.software.informer.com/15.3/).

**Fig. 5:**
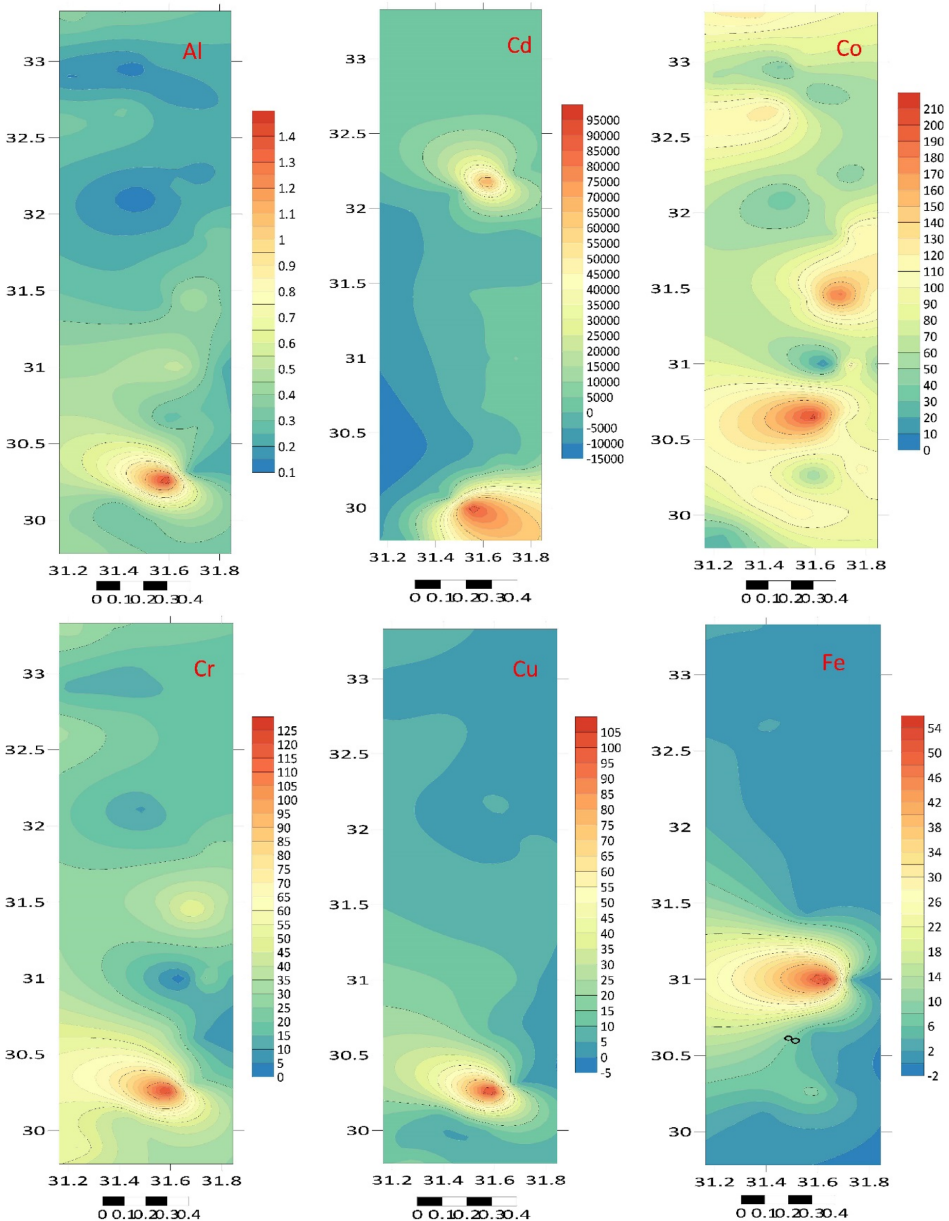

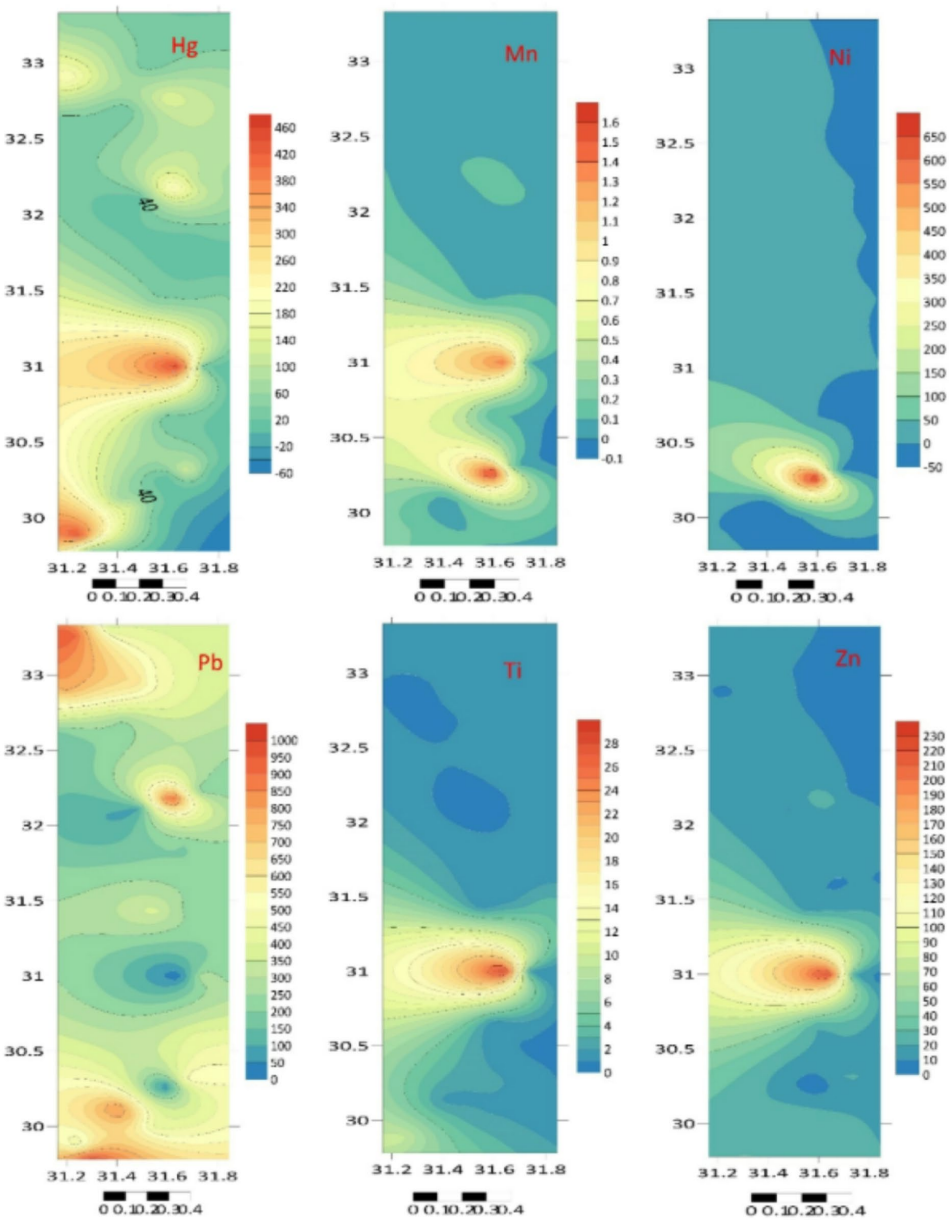
The enrichment factor (EF) distributions in the Nile Delta coastal area sediment samples (Surfer 15.3 software: https://surfer.software.informer.com/15.3/).

Another frequently used technique to evaluate trace metal pollution in the sediments of the current study is the *I*_geo_, which was first presented by Muller^59^. Metal contamination in sediments is analyzed and described using the *I*_geo_^59^. Table 2; Fig. 4 provide an overview of the calculated *I*_geo_ for the sediments. Overall, according to Tucker’s scale, HM pollution throughout the Egyptian Mediterranean coast has not been significant, which shows that the mean *I*_geo_ of these metals is less than zero (*I*_geo_ < 0). The *EF* was computed to assess the anthropogenic effect, and the results are shown in Table 3; Fig. 5^49,50^.

The range value of Zn ranged from 1.14 to 231.82, Pb ranged from 0.95 to 980.43, Cd ranged from 73.48 to 79189.11, Mn ranged from 0.03 to 1.44, and Cu ranged from 2.23 to 113.63.

**Table 2:** The *I*_geo_ in the nile Delta coastal area sediments.

Location	Al	Ti	Cr	Mn	Fe	Co	Ni	Cu	Zn	Cd	Hg	Pb
A2	− 10.29	− 5.80	− 3.25	− 11.25	− 9.70	− 4.17	− 9.33	− 5.78	− 4.73	− 1.73	− 0.26	− 0.25
A3	− 10.22	− 6.20	− 3.61	− 11.46	− 9.10	− 4.06	− 6.95	− 4.92	− 4.08	− 1.33	− 3.74	0.96
B1	− 10.55	− 8.33	− 3.61	− 12.09	− 8.35	− 2.13	− 7.39	− 5.48	− 4.62	− 1.15	− 2.87	0.63
B2	− 10.65	− 7.83	− 3.78	− 12.49	− 8.02	− 2.22	− 8.46	− 5.75	− 4.37	0.20	− 2.07	0.48
B3	− 10.52	− 7.84	− 3.85	− 11.49	− 8.24	− 2.17	− 8.36	− 6.21	− 4.16	7.47	− 4.47	− 0.31
C1	− 10.41	− 8.40	− 4.33	− 12.33	− 7.78	− 2.00	− 8.28	− 5.82	− 3.52	0.73	− 2.84	− 0.11
C2	− 11.28	− 8.52	− 4.22	− 12.28	− 8.88	− 2.61	− 8.39	− 6.55	− 4.73	3.02	− 2.05	0.02
C3	− 10.93	− 8.43	− 4.18	− 12.68	− 8.39	− 2.20	− 8.11	− 6.27	− 4.67	− 0.90	− 4.91	− 0.05
D1	− 10.91	− 8.99	− 4.00	− 12.51	− 7.48	− 1.30	− 8.27	− 5.97	− 4.69	− 1.36	− 4.77	− 0.71
D2	− 10.50	− 8.49	− 5.74	− 12.72	− 7.63	− 2.47	− 8.28	− 6.29	− 4.60	− 0.58	− 4.47	− 0.34
E1	− 11.82	− 9.33	− 4.16	− 13.27	− 8.51	− 3.24	− 8.79	− 7.03	− 5.35	− 0.04	− 4.77	− 0.20
E2	− 10.49	− 8.52	− 4.33	− 12.85	− 8.24	− 2.25	− 8.71	− 6.36	− 4.90	3.45	− 4.42	− 0.86
E3	− 11.43	− 8.76	− 5.45	− 13.73	− 9.58	− 3.69	− 9.47	− 7.88	− 6.15	0.06	− 5.91	− 1.19
F1	− 11.03	− 8.71	− 3.99	− 13.15	− 8.56	− 2.6	− 8.77	− 6.49	− 5.10	− 1.04	− 3.53	− 0.49
F2	− 10.13	− 7.89	− 3.33	− 12.39	− 7.57	− 1.43	− 8.08	− 6.09	− 5.13	− 1.30	− 3.94	− 1.23
F3	− 10.64	− 7.20	− 3.94	− 12.73	− 8.17	− 2.17	− 9.11	− 6.41	− 4.90	− 1.26	− 3.56	− 1.33
G1	− 11.25	− 8.85	− 4.69	− 13.57	− 8.63	− 3.17	− 9.07	− 6.87	− 5.42	− 1.57	− 5.23	− 1.42
G2	− 10.25	− 8.68	− 3.94	− 12.54	− 7.83	− 1.75	− 8.68	− 6.09	− 5.05	− 0.91	− 0.89	− 1.75
G3	− 10.70	− 8.61	− 4.35	− 12.81	− 8.22	− 2.12	− 9.01	− 6.44	− 4.90	− 0.60	− 4.47	− 1.14
H1	− 12.22	− 10.02	− 5.93	− 14.05	− 9.88	− 4.08	− 10.23	− 7.32	− 5.78	− 0.99	− 6.23	− 2.55
H2	− 11.30	− 9.42	− 4.75	− 11.22	− 8.87	− 2.90	− 8.64	− 6.44	− 4.31	7.23	− 1.22	0.90
H3	− 11.47	− 8.17	− 5.00	− 13.27	− 9.61	− 3.65	− 10.16	− 7.77	− 5.97	3.74	− 2.53	− 1.23
I1	− 10.68	− 8.82	− 4.05	− 12.81	− 8.13	− 2.14	− 8.79	− 6.56	− 5.42	1.81	− 4.42	− 1.48
I2	− 10.73	− 9.22	− 4.51	− 12.67	− 7.93	− 2.00	− 8.59	− 6.43	− 5.11	1.25	− 4.77	− 1.14
I3	− 11.49	− 8.26	− 5.00	− 13.41	− 9.17	− 3.71	− 9.08	− 7.71	− 6.02	0.42	− 1.90	− 0.40
J1	− 11.87	− 9.31	− 5.33	− 13.36	− 9.11	− 3.21	− 7.47	− 7.30	− 5.87	0.90	− 1.36	0.54
J2	− 12.10	− 8.75	− 5.75	− 13.27	− 9.27	− 4.07	− 7.20	− 7.18	− 5.55	1.37	− 3.64	0.28
J3	− 11.31	− 8.46	− 5.01	− 13.14	− 9.01	− 2.76	− 8.40	− 7.06	− 5.96	0.91	− 4.37	− 0.04
K1	− 10.50	− 8.52	− 4.07	− 12.68	− 8.03	− 2.18	− 6.71	− 6.28	− 4.72	2.97	− 3.45	0.84
K2	− 10.68	− 8.88	− 4.39	− 12.77	− 7.99	− 2.05	− 8.09	− 6.57	− 5.14	1.57	− 3.23	− 0.62
K3	− 10.52	− 8.56	− 4.34	− 12.78	− 8.10	− 2.15	− 8.23	− 6.52	− 5.24	− 0.50	− 4.27	− 0.42

**Table 3:** The enrichment factor (*EF*) in the nile Delta coastal area sediment samples.

Location	Al	Ti	Cr	Mn	Fe	Co	Ni	Cu	Zn	Cd	Hg	Pb
A2	0.42	9.46	55.39	0.22	0.63	29.23	0.82	9.62	19.85	159.32	439.13	442.66
A3	0.44	7.16	43.17	0.19	0.96	31.54	4.27	17.38	31.12	209.69	39.36	1027.07
B1	0.35	1.64	43.04	0.12	1.61	120.34	3.14	11.83	21.47	237.81	71.73	817.82
B2	0.33	2.32	38.30	0.09	2.03	113.48	1.49	9.79	25.58	606.82	125.09	735.92
B3	0.36	2.30	36.66	0.18	1.74	117.51	1.60	7.11	29.54	93367.42	23.62	424.63
C1	1.56	2.40	132.23	1.70	9.33	46.08	717.18	113.63	1.14	73.48	0.39	2.26
C2	0.21	1.43	28.27	0.11	1.12	86.20	1.57	5.64	19.85	4262.98	126.84	535.47
C3	0.27	1.53	29.00	0.08	1.57	114.37	1.91	6.84	20.73	282.32	17.50	510.64
D1	0.27	1.04	32.86	0.09	2.95	213.84	1.71	8.41	20.46	205.01	19.24	322.10
D2	0.36	1.46	9.88	0.08	2.65	95.41	1.70	6.73	21.67	352.61	23.62	415.16
E1	0.53	29.47	0.05	1.44	55.86	1.19	4.03	12.94	231.82	295.21	460.41	0.95
E2	0.37	1.44	26.22	0.07	1.74	110.56	1.26	6.42	17.63	5749.58	24.49	290.15
E3	0.19	1.22	12.08	0.04	0.69	40.82	0.74	2.23	7.42	550.59	8.75	230.46
F1	0.25	1.25	33.13	0.06	1.40	86.79	1.20	5.88	15.40	256.55	45.49	375.04
F2	0.47	2.22	52.27	0.10	2.77	195.01	1.95	7.74	15.03	214.38	34.12	224.01
F3	0.33	3.60	34.43	0.08	1.83	116.83	0.95	6.20	17.66	220.24	44.61	209.16
G1	0.22	1.14	20.36	0.04	1.33	58.49	0.98	4.51	12.32	178.06	14.00	196.75
G2	0.22	1.14	20.36	0.04	1.33	58.49	0.98	4.51	12.32	178.06	14.00	196.75
G3	0.32	1.35	25.79	0.07	1.77	121.30	1.02	6.05	17.61	346.76	23.62	239.02
H1	0.11	0.51	8.66	0.03	0.56	31.26	0.44	3.30	9.56	265.92	7.00	90.27
H2	0.21	0.77	19.60	0.22	1.13	70.44	1.32	6.08	26.53	79189.11	224.81	980.43
H3	0.19	1.83	16.49	0.05	0.68	41.88	0.46	2.42	8.38	7055.77	90.98	224.68
I1	0.32	1.16	31.88	0.07	1.88	119.32	1.19	5.60	12.30	1849.75	24.49	189.64
I2	0.31	0.88	23.06	0.08	2.17	131.86	1.36	6.10	15.28	1258.16	19.24	238.84
I3	0.18	1.72	16.53	0.05	0.91	40.30	0.97	2.52	8.11	704.05	140.84	399.83
J1	0.14	0.83	13.12	0.05	0.96	56.99	2.97	3.35	9.01	981.69	203.82	766.30
J2	0.12	1.22	9.81	0.05	0.86	31.46	3.60	3.64	11.23	1360.08	41.99	642.07
J3	0.21	1.49	16.33	0.06	1.02	77.56	1.56	3.95	8.48	987.55	25.37	514.40
K1	0.36	1.44	31.41	0.08	2.02	116.48	5.04	6.79	19.95	4120.06	48.11	945.18
K2	0.32	1.12	25.10	0.08	2.08	127.41	1.93	5.54	15.00	1560.40	55.98	342.15
K3	0.36	1.40	26.07	0.07	1.92	119.01	1.76	5.73	13.99	372.53	27.12	394.21

**Fig. 6:**
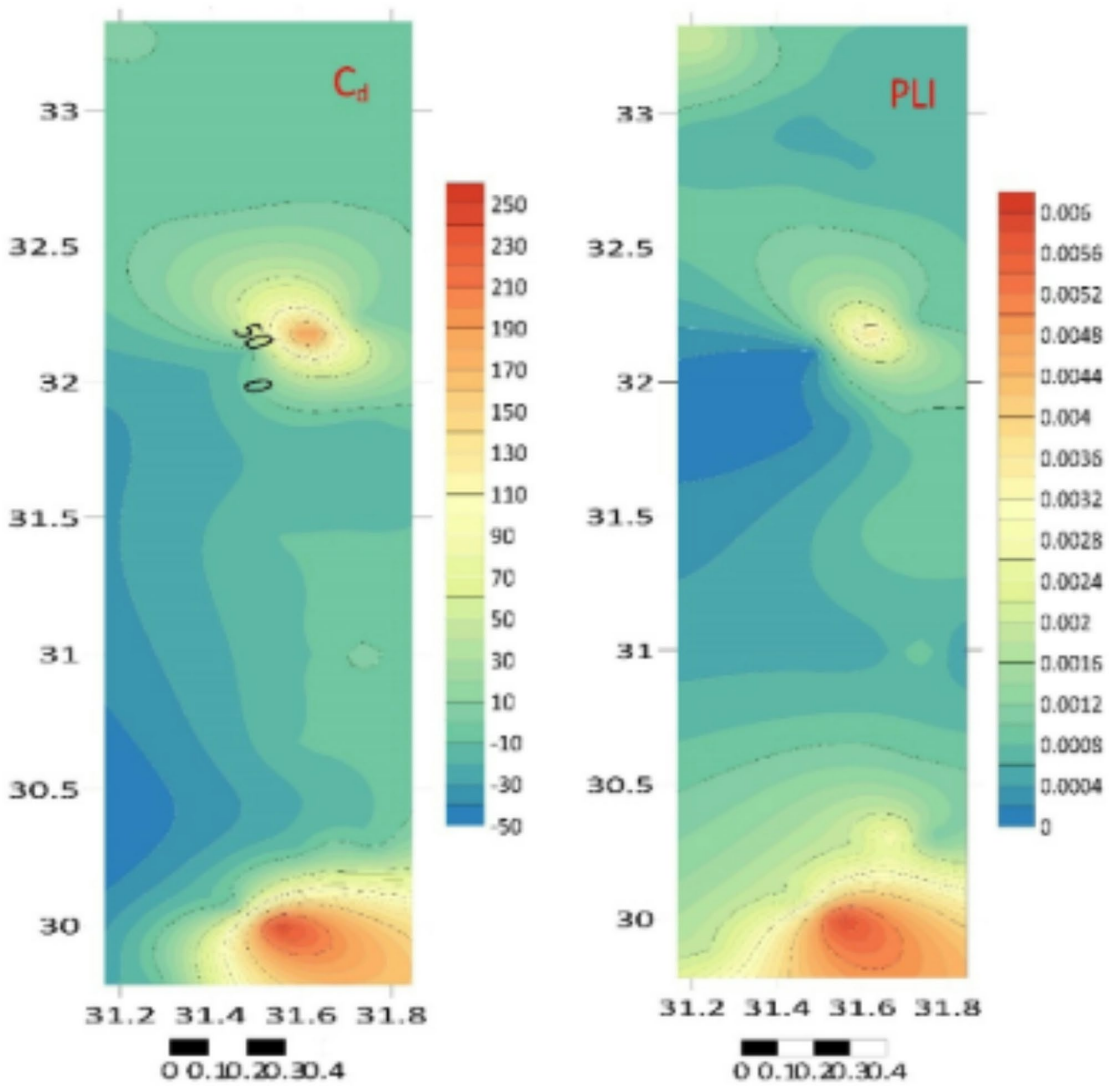
The distributions of the PLI and contamination degree in the Egyptian Mediterranean coast sediment samples (Surfer 15.3 software: https://surfer.software.informer.com/15.3/).

Table 4; Fig. 6 show that the *PLI* measurements ranged from 0.0001 to 0.006, suggesting no coastal silt had been affected^51^.

Table 4; Fig. 6 show that the values of Cd ranged from − 10.81 to 255.5^52^. The CD provides a comprehensive assessment of pollution that considers many PTEs. PLI offers a comprehensive analysis that considers various metal toxicities, EF assesses regulatory compliance directly, and *I*_geo_ offers a straightforward assessment. The indicators will depend on the legal framework and the specific evaluation objectives.

**Table 4:** The *PLI* and *C*_d_ levels were determined in the nile Delta coastal area sediment samples.

Location	PLI	C_d_
A2	0.002	− 8.46
A3	0.003	− 7.98
B1	0.002	− 8.21
B2	0.002	− 7.27
B3	0.006	255.5
C1	0.002	− 7.29
C2	0.003	2.43
C3	0.001	− 9.19
D1	0.001	− 9.64
D2	0.001	− 9.35
E1	0.0005	− 8.88
E2	0.001	5.72
E3	0.0003	− 9.57
F1	0.0007	− 9.66
F2	0.001	− 9.87
F3	0.001	− 10.13
G1	0.0003	− 10.61
G2	0.001	− 9.32
G3	0.0009	− 9.77
H1	0.0001	− 10.81
H2	0.004	217.1
H3	0.0009	9.18
I1	0.0009	− 5.63
I2	0.0009	− 7.17
I3	0.0006	− 8.25
J1	0.0006	− 6.19
J2	0.0005	− 6.01
J3	0.0007	− 7.34
K1	0.002	3.07
K2	0.001	− 5.92
K3	0.0009	− 9.26

**Fig. 7:**
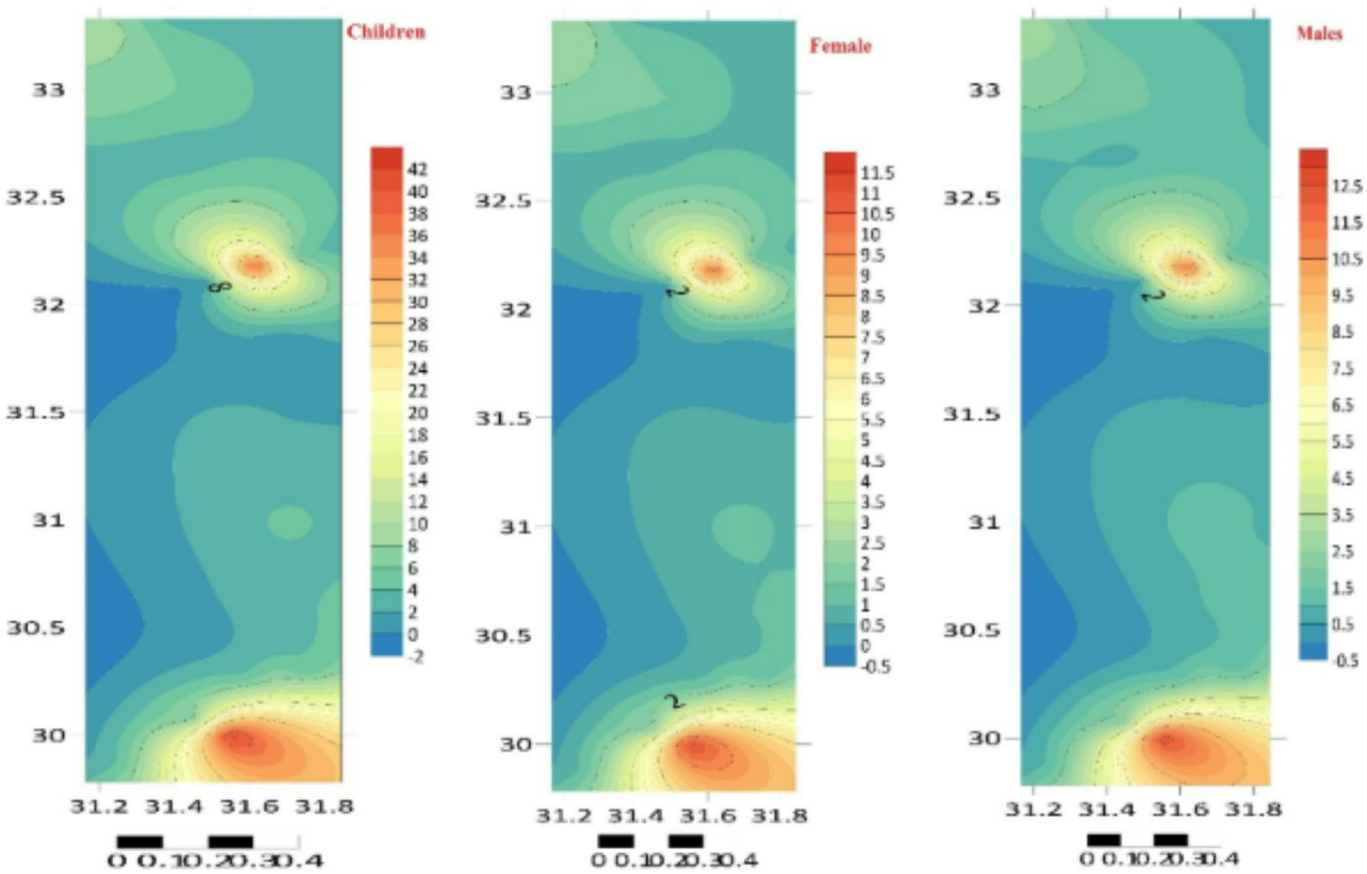
The total hazard index (THI) of the 25 elements detected (µg/g dry weight) in the sediment at the study sites, distributed spatially (Surfer 15.3 software: https://surfer.software.informer.com/15.3/).

**Fig. 8:**
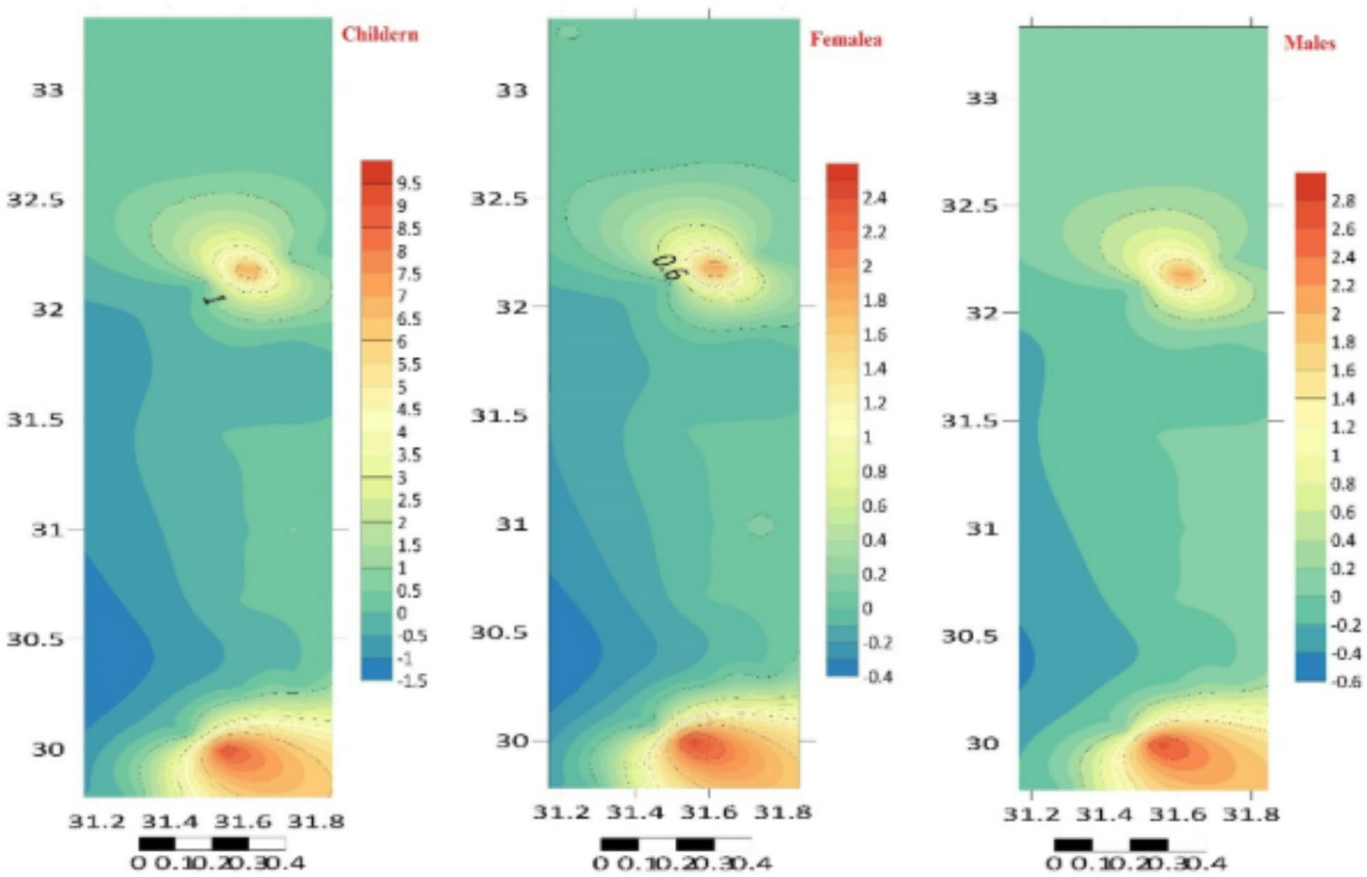
The overall carcinogenic risk (TCR) of the 25 elements detected (µg/g dry weight) in the sediment at the study sites, distributed spatially (Surfer 15.3 software: https://surfer.software.informer.com/15.3/).

To determine *CDI*, *CR*, *HQ*, and *HI*, the dermal adsorption approach was used to compute the human risk assessment for children, females, and males (carcinogenic and non-carcinogenic). For children, girls, and men, the *CDI* (mg kg^–1^ Day^–1^) values were given in Tables 3, 4 and 5 S. Zn < Cu < Pb < Mn < Fe were the PTEs based on *CDI* readings (Tables S4–S6). Table S7 and Fig. 7 present the *HQ* and *THI* values for the different groups, which indicate that children have a low value compared to the adult population, which is three to four times greater. The lower *HQ* and *HI* values for children than adults are likely due to methodological factors in the risk assessment process, including normalization for body weight, protective assumptions, and variations in exposure scenarios. In terms of PTEs, humans were grouped according to demand hierarchy: Cd > Cu > Fe > Mn > Pb > Zn. The cutaneous route was used to assess the *CR* of Pb and Cd because of their carcinogenic slope factor. The ranges of *CR* values for *C*_d_ indicated a higher risk of cancer (> 10–6): 9.78 × 10–4–1.52 × 10^–1^ for men, 9.35 × 10^–4^ – 1.34 × 10^–1^ for females, and 9.99 × 10^–4^ – 4.97 × 10^–1^ for children^56,60–62^. The carcinogenic risk (*CR*) for lead (Pb) varies depending on the gender and age group: women (5.79 × 10^–6^ – 6.59 × 10^–5^), males (6.51 × 10^–6^ – 7.41 × 10^–5^), and children (3.72 × 10^–5^ – 2.43 × 10^–4^) (Tables S7, S8, S9 and Fig. 8).

The eco-toxicological significance of HM contamination in sediment was ascertained using sediment quality criteria created for marine and estuarine environments^63^. These effects are the effect range low (*ERL*)/effect range median (*ERM*) and the threshold effect level (*TEL*)/probable effect level (*PEL*), as shown in Table 5. While *ERM* and *PEL* reveal chemical concentrations above the anticipated prevalence of negative effects, *ERL* and *TEL* show chemical concentrations below the harmful consequences upon species living in sediment. There have been reports of both a probable effect concentration (PEC), which is the concentration at which toxicity is frequently recognized, and a threshold effect concentration (TEC), which is the value at which toxicity may be seen^64^. Sediment pollution may be evaluated by comparing several indications of contamination with effect-based sediment quality guidelines (*SQGs*) for Cu, Zn and Ni only. Numerical *SQGs* have been utilized in aquatic environments to identify contaminants of concern^64^ (Table 5). Sediments were classified as non-contaminated, moderately polluted, or very polluted based on USEPA *SQGs*^65^ (Table 5). The examined sediments are not contaminated with Cu, Zn, or Ni by *SQG* criteria; however, the amounts of Cd are more significant than those of *PEL* and *ERM* (Table 5).

**Table 5:** *SQGs* and HM concentrations (µg/g dry weight) are evaluated using many criteria.

Metals	This study (average concentrations)	Shale	SQG			PEL	TEL	ERL	ERM
			Non-polluted	Moderate polluted	Heavily polluted				
Cu	12.66	45	> 25	25–50	> 50	110	18.7	34	270
Cd	125.95	0.30	–	–	–	4.2	0.68	1.2	9.6
Zn	10.76	95	> 90	90–200	> 200	270	124	150	410
Co	8.18	19	–	–	–	–	–	–	–
Ni	1.38	68	> 20	20–50	> 50	43	15.9	20.9	51.6
Mn	1.80	850	–	–	–	–	–	–	–
Fe	394.46	47,600	–	–	–	–	–	–	–
Al	345.03	80,000	–	–	–	–	–	–	–

### Statistical analysis

#### PTEs correlation coefficient analysis

Pearson’s correlation matrix assessed the association structure between the identified metals. Table S10 displays the strong relationships found in the correlation matrix study between Ti and Ag (*R* = 0.978), Ca and Cu (*R* = 0.898), K and Co (*R* = 0.891), Fe and Co (*R* = 0.912), and the moderate correlations between B and Ti (*R* = 0.9), B and Ag (*R* = 0.924), Na and Cu (*R* = 0.781), Al and Cr (*R* = 0.781), Al and Cu (*R* = 0.718), K and Fe (*R* = 0.868), Cr and Cu (*R* = 0.709), and Cd and Bi (*R* = 0.912), suggest that these metals may share similar sources, undergo co-precipitation, or exhibit linked behavior in sedimentary environments, reflecting complex geochemical interactions and environmental processes^66,67^. Other correlations between metals in sediments, such as Mn and Cu (0.650), Zn (0.631), and Se (0.591). Al and Ti, Cr, Mn, and Fe have good connections (*R* = 0.467, 0.723, 0.482, and 0.611, respectively) are also observed. The strong correlation between Cu and Zn (*R* = 0.692) confirms earlier findings that they co-occur in minerals that form rocks and have similar geochemical behavior^66,67^.

#### Principal component analysis (PCA)

The *PCA* is a powerful method for problems related to pattern recognition, classification, modelling, and other data assessment (Table S11 and Fig. S1)^68,69^, and it’s between the initial and greatest straightforward techniques for analyzing common factors. Since these components are orthogonal linear combinations of measurement variables, they may help compare or analyze the properties of the 25 elements found in the sediments at the locations under study. These elements are referred to as axes. When the ordination function’s first two axes are displayed, data from an experimental system with comparable characteristics are plotted closely together, and data with differences are presented widely apart^70–74^. With a high loading of Ca, Na, Ga, Cu, and Ti, the first group (PC1) accounted for 33.398% of the total variance, indicating that these metals were associated throughout the process (Table S11). There are common source pieces for these metals. With a high loading of Cd (0.910), the second component (PC2) accounted for 20.974% of the overall variation. With a percentage of 18.529%, the third component (PC3) correlated 0.936 with Co and 0.932 with Fe.

The variations in metal concentrations between the Mediterranean Sea and the samples collected for this investigation are shown in Table S12. It is noteworthy that the Mn concentrations measured are lower than those found in samples from the coasts of Rosetta^75^, Abu-Qir Bay^76^, the lagoons of Manzala and Edku^77^, Egypt’s Mediterranean coast^78,79^, Libya’s Mediterranean coast^80^, Morocco’s coasts^81^, and Safax, Tunisia^82^. Comparable to any previously named locations, there are lower amounts of Cu, Cd and Zn. The Pb levels are greater than those of the Mediterranean coast between Port Said and Abu-Qir Bay, Egypt, but lower than those of the Rosetta coast and Manzala Lagoon near North Carolina. Accordingly, higher Pb concentrations near their Mediterranean coast may result from Egypt, Tunisia, and Morocco’s rapid industrialization.

## Conclusion

The present communication reports on a primary investigation conducted on surface sediments of the Nile Delta coastal area. The study includes measurements of levels of 25 different elements. Thirty-one sediment samples were gathered from eleven sampling sectors along the shore to examine the possibility of element contamination in the sediment samples resulting from the numerous activities along Egypt’s eastern Mediterranean coast. The average concentration of all elements was 49.86 ± 99.44 µg/g. With a value of 208.86 ± 788.71 µg/g, A3 Station had the highest average value in this research because of the numerous industrial activities and factories nearby. This study also showed that the mean *I*_geo_ of these metals is less than zero (*I*_geo_ <0), suggesting the Nile Delta coastal area has not been substantially polluted by PTEs.

Furthermore, no polluted coastal silt was found, as shown by the *PLI* measurements, which ranged from 0.0001 to 0.006. According to the correlation matrix analysis, there are strong relationships between Mn and Cu, Zn and Se, and Al and Ti, Cr, Mn, and Fe, in that order. Future research should focus on long-term monitoring programs to assess temporal trends in HM concentrations and their ecological impacts. Advanced geochemical modelling and source apportionment studies could provide deeper insights into contamination sources. Policymakers are encouraged to develop stricter regulations on industrial discharges and enhance wastewater treatment protocols to mitigate potential risks. Moreover, implementing mitigation strategies such as promoting sustainable industrial practices, rehabilitating polluted hotspots, and improving public awareness about pollution prevention could further safeguard the region’s coastal and marine ecosystems. These measures, combined with robust monitoring and enforcement mechanisms, are crucial for maintaining the ecological integrity of the Nile Delta’s coastal zone and ensuring the sustainable use of its resources.

## Electronic supplementary material

Below is the link to the electronic supplementary material.

Supplementary Material 1

## Data Availability

Data will be available upon request from the corresponding author.
