# Supplementary material for: Elements pollution and ecological risk assessment of coastal sediments along the Nile Delta
Source: Sci Rep. 2025 Jun 20;15:20222. doi: 10.1038/s41598-025-06801-4 (PMC12181402; doi:10.1038/s41598-025-06801-4)
Supplement: Supplementary file 1 — Supplementary Material 1 [file 41598_2025_6801_MOESM1_ESM.docx]

Supplementary data

**Elements pollution and ecological risk assessment of coastal sediments along the Nile Delta**

Mohamed A. Hassaan^1^, Amr G. Dardeer^2^, Ahmed El Nemr^1*^

^1^National Institute of Oceanography and Fisheries (NIOF), Kayet Bey, El-Anfoushy, Alexandria, Egypt

^2^Administration of Environmental Monitoring - Ministry of Health and Population, Alexandria, Egypt.

Email: [mhss95@mail.com](mailto:mhss95@mail.com) (M.A. Hassaan); [amrbiochemistry@gmail.com](mailto:amrbiochemistry@gmail.com) (A.G. Dardeer);

*Corresponding author Email: [ahmedmoustafaelnemr@yahoo.com](mailto:ahmedmoustafaelnemr@yahoo.com); [ahmed.m.elnemr@gmail.com](mailto:ahmed.m.elnemr@gmail.com)

Table S1. The sample stations are described below (Depth and geographic location).

| **Location** | **Long** | **Lat** | **Depth** |
| --- | --- | --- | --- |
| A2 | 29° 52.86' | 31° 14.88' | 30 |
| A3 | 29° 46.8' | 31° 17.46' | 50 |
| B1 | 31**°** 24.57**'** | 30**°** 06.8**'** | 10 |
| B2 | 30° 4.5' | 31° 27' | 30 |
| B3 | 30° 0.3' | 31° 32.58' | 50 |
| C1 | 30° 15.84' | 31° 35.58' | 10 |
| C2 | 30° 18.66' | 31° 40.68' | 30 |
| C3 | 30° 17.94' | 31° 45.36' | 50 |
| D1 | 30° 39.36' | 31° 35.58' | 10 |
| D2 | 30° 36.12' | 31° 42.78' | 30 |
| E1 | 30° 59.82' | 31° 38.82' | 10 |
| E2 | 30° 59.58' | 31° 44.16' | 30 |
| E3 | 30° 59.34' | 31° 50.7' | 50 |
| F1 | 31° 25.86' | 31° 32.82' | 10 |
| F2 | 31° 27.06' | 31° 41.16' | 30 |
| F3 | 31° 27.24' | 31° 48.84' | 50 |
| G1 | 31° 50.52' | 31° 33.72' | 10 |
| G2 | 31° 42.7' | 31° 52.5' | 30 |
| G3 | 31° 52.38' | 31° 45.12' | 50 |
| H1 | 32° 6.54' | 31° 29.28' | 10 |
| H2 | 32° 10.5' | 31° 36.96' | 25 |
| H3 | 32° 13.8' | 31° 43.26' | 35 |
| I1 | 31° 12.9' | 32°42.06' | 10 |
| I2 | 32° 41.16' | 31° 25.8' | 30 |
| I3 | 31° 25.7' | 32° 41.3' | 50 |
| J1 | 32° 54.24' | 31° 12.48' | 10 |
| J2 | 32° 57.78' | 31° 28.74' | 30 |
| J3 | 32° 59.34' | 31° 31.92' | 50 |
| K1 | 33° 16.2' | 31° 13.56' | 10 |
| K2 | 33° 19.32' | 31° 22.8' | 30 |
| K3 | 33° 19.74' | 31° 24.24' | 50 |

Table S2. Analytical results of certified and obtained values (ppm) of elements in standard reference materials SRM 2702.

| **Element** | Certified | **SRM 2702** |  |
| --- | --- | --- | --- |
|  |  | Measured | Recovery (%) |
| Li | 78.2 | 77.2 | 98.7 |
| Na | 0.681* | 0.685 | 100.6 |
| Mg | 0.990* | 0.999 | 100.9 |
| Al | 8.41* | 8.30 | 98.7 |
| K | 2.054* | 2.001 | 97.4 |
| Ca | 0.343* | 0.335 | 97.7 |
| Ti | 0.884* | 0.879 | 99.4 |
| Cr | 352 | 347 | 98.6 |
| Mn | 1757 | 1732 | 98.6 |
| Fe | 7.91* | 7.70 | 97.3 |
| Co | 27.76 | 27.45 | 98.9 |
| Ni | 75.4 | 74.7 | 99.1 |
| Cu | 117.7 | 114.5 | 97.6 |
| Zn | 485.3 | 478.5 | 98.6 |
| Ga | 24.3 | 23.6 | 97.1 |
| Se | 4.95 | 4.84 | 97.7 |
| Sr | 119.7 | 118.5 | 99.0 |
| Ag | 0.622 | 0.619 | 99.7 |
| Cd | 0.817 | 0.814 | 99.6 |
| Ba | 397.4 | 396.1 | 99.7 |
| Pb | 132.8 | 131.5 | 99.0 |

*Percentage (%) was used as the measurement unit of the major elements (Na, Mg, Al, K, Ca, Ti and Fe).

Table S3. Reference dose (dermal) and slope factor for different PTEs.

| **Parameters** | **Definitions** | **Cd** | **Cu** | **Mn** | **Fe** | **Pb** | **Zn** | **References** |
| --- | --- | --- | --- | --- | --- | --- | --- | --- |
| RfD_Dermal_ (mg/kg/day) | Reference dose | 1x10^-3^ | 4x10^-2^ | 1.4x10^-1^ | 0.8 | 3.52x10^-3^ | 0.3 | [1] |
| SF_dermal_  (mg/kg/day) | Slope factor | 15 | - | - | - | 8.5x10^-3^ | - |  |

Table S4. The Chronic Daily Intake (CDI) for dermal absorption in males.

| **Label** | **Al** | **Ti** | **Cr** | **Mn** | **Fe** | **Co** | **Ni** | **Cu** | **Zn** | **Cd** |  | **Hg** | **Pb** |
| --- | --- | --- | --- | --- | --- | --- | --- | --- | --- | --- | --- | --- | --- |
| **A2** | 1.42E-02 | 1.85E-02 | 2.12E-03 | 7.79E-05 | 1.27E-02 | 2.36E-04 | 2.37E-05 | 1.84E-04 | 8.00E-04 | 2.03E-05 |  | 7.49E-05 | 3.76E-03 |
| **A3** | 1.50E-02 | 1.40E-02 | 1.65E-03 | 6.74E-05 | 1.93E-02 | 2.54E-04 | 1.23E-04 | 3.32E-04 | 1.25E-03 | 2.67E-05 |  | 6.71E-06 | 8.72E-03 |
| **B1** | 1.19E-02 | 3.20E-03 | 1.64E-03 | 4.35E-05 | 3.23E-02 | 9.70E-04 | 9.05E-05 | 2.26E-04 | 8.65E-04 | 3.03E-05 |  | 1.22E-05 | 6.94E-03 |
| **B2** | 1.11E-02 | 4.53E-03 | 1.46E-03 | 3.30E-05 | 4.08E-02 | 9.15E-04 | 4.31E-05 | 1.87E-04 | 1.03E-03 | 7.73E-05 |  | 2.13E-05 | 6.25E-03 |
| **B3** | 1.21E-02 | 4.49E-03 | 1.40E-03 | 6.61E-05 | 3.49E-02 | 9.48E-04 | 4.62E-05 | 1.36E-04 | 1.19E-03 | 1.19E-02 |  | 4.03E-06 | 3.60E-03 |
| **C1** | 1.31E-02 | 3.04E-03 | 1.00E-03 | 3.70E-05 | 4.82E-02 | 1.07E-03 | 4.89E-05 | 1.78E-04 | 1.86E-03 | 1.11E-04 |  | 1.25E-05 | 4.16E-03 |
| **C2** | 7.19E-03 | 2.80E-03 | 1.08E-03 | 3.82E-05 | 2.24E-02 | 6.95E-04 | 4.53E-05 | 1.08E-04 | 8.00E-04 | 5.43E-04 |  | 2.16E-05 | 4.54E-03 |
| **C3** | 9.20E-03 | 2.99E-03 | 1.11E-03 | 2.89E-05 | 3.15E-02 | 9.22E-04 | 5.52E-05 | 1.31E-04 | 8.36E-04 | 3.59E-05 |  | 2.98E-06 | 4.33E-03 |
| **D1** | 9.32E-03 | 2.02E-03 | 1.25E-03 | 3.27E-05 | 5.90E-02 | 1.72E-03 | 4.92E-05 | 1.61E-04 | 8.25E-04 | 2.61E-05 |  | 3.28E-06 | 2.73E-03 |
| **D2** | 1.24E-02 | 2.85E-03 | 3.77E-04 | 2.82E-05 | 5.31E-02 | 7.69E-04 | 4.89E-05 | 1.29E-04 | 8.74E-04 | 4.49E-05 |  | 4.03E-06 | 3.52E-03 |
| **E1** | 4.96E-03 | 1.60E-03 | 1.13E-03 | 1.92E-05 | 2.89E-02 | 4.50E-04 | 3.43E-05 | 7.70E-05 | 5.22E-04 | 6.52E-05 |  | 3.28E-06 | 3.91E-03 |
| **E2** | 1.24E-02 | 2.81E-03 | 1.00E-03 | 2.58E-05 | 3.48E-02 | 8.91E-04 | 3.64E-05 | 1.23E-04 | 7.11E-04 | 7.32E-04 |  | 4.18E-06 | 2.46E-03 |
| **E3** | 6.49E-03 | 2.38E-03 | 4.61E-04 | 1.40E-05 | 1.38E-02 | 3.29E-04 | 2.15E-05 | 4.27E-05 | 2.99E-04 | 7.01E-05 |  | 1.49E-06 | 1.96E-03 |
| **F1** | 8.53E-03 | 2.45E-03 | 1.27E-03 | 2.09E-05 | 2.81E-02 | 7.00E-04 | 3.48E-05 | 1.12E-04 | 6.21E-04 | 3.27E-05 |  | 7.76E-06 | 3.18E-03 |
| **F2** | 1.60E-02 | 4.34E-03 | 2.00E-03 | 3.55E-05 | 5.55E-02 | 1.57E-03 | 5.64E-05 | 1.48E-04 | 6.06E-04 | 2.73E-05 |  | 5.82E-06 | 1.90E-03 |
| **F3** | 1.12E-02 | 7.02E-03 | 1.32E-03 | 2.80E-05 | 3.67E-02 | 9.42E-04 | 2.74E-05 | 1.18E-04 | 7.12E-04 | 2.80E-05 |  | 7.61E-06 | 1.78E-03 |
| **G1** | 7.33E-03 | 2.23E-03 | 7.77E-04 | 1.57E-05 | 2.67E-02 | 4.72E-04 | 2.83E-05 | 8.62E-05 | 4.97E-04 | 2.27E-05 |  | 2.39E-06 | 1.67E-03 |
| **G2** | 1.47E-02 | 2.51E-03 | 1.31E-03 | 3.19E-05 | 4.65E-02 | 1.26E-03 | 3.70E-05 | 1.48E-04 | 6.43E-04 | 3.58E-05 |  | 4.83E-05 | 1.33E-03 |
| **G3** | 1.07E-02 | 2.63E-03 | 9.85E-04 | 2.64E-05 | 3.55E-02 | 9.78E-04 | 2.95E-05 | 1.16E-04 | 7.10E-04 | 4.41E-05 |  | 4.03E-06 | 2.03E-03 |
| **H1** | 3.75E-03 | 9.90E-04 | 3.31E-04 | 1.12E-05 | 1.12E-02 | 2.52E-04 | 1.27E-05 | 6.29E-05 | 3.86E-04 | 3.39E-05 |  | 1.19E-06 | 7.66E-04 |
| **H2** | 7.10E-03 | 1.50E-03 | 7.49E-04 | 7.98E-05 | 2.26E-02 | 5.68E-04 | 3.82E-05 | 1.16E-04 | 1.07E-03 | 1.01E-02 |  | 3.83E-05 | 8.32E-03 |
| **H3** | 6.33E-03 | 3.57E-03 | 6.30E-04 | 1.92E-05 | 1.36E-02 | 3.38E-04 | 1.33E-05 | 4.62E-05 | 3.38E-04 | 8.98E-04 |  | 1.55E-05 | 1.91E-03 |
| **I1** | 1.09E-02 | 2.27E-03 | 1.22E-03 | 2.65E-05 | 3.77E-02 | 9.62E-04 | 3.43E-05 | 1.07E-04 | 4.96E-04 | 2.35E-04 |  | 4.18E-06 | 1.61E-03 |
| **I2** | 1.05E-02 | 1.72E-03 | 8.81E-04 | 2.92E-05 | 4.34E-02 | 1.06E-03 | 3.94E-05 | 1.16E-04 | 6.16E-04 | 1.60E-04 |  | 3.28E-06 | 2.03E-03 |
| **I3** | 6.23E-03 | 3.35E-03 | 6.31E-04 | 1.74E-05 | 1.83E-02 | 3.25E-04 | 2.80E-05 | 4.82E-05 | 3.27E-04 | 8.96E-05 |  | 2.40E-05 | 3.39E-03 |
| **J1** | 4.77E-03 | 1.62E-03 | 5.01E-04 | 1.80E-05 | 1.91E-02 | 4.60E-04 | 8.58E-05 | 6.40E-05 | 3.63E-04 | 1.25E-04 |  | 3.48E-05 | 6.50E-03 |
| **J2** | 4.08E-03 | 2.38E-03 | 3.75E-04 | 1.92E-05 | 1.71E-02 | 2.54E-04 | 1.04E-04 | 6.95E-05 | 4.53E-04 | 1.73E-04 |  | 7.16E-06 | 5.45E-03 |
| **J3** | 7.05E-03 | 2.92E-03 | 6.24E-04 | 2.10E-05 | 2.05E-02 | 6.25E-04 | 4.49E-05 | 7.55E-05 | 3.42E-04 | 1.26E-04 |  | 4.33E-06 | 4.37E-03 |
| **K1** | 1.24E-02 | 2.80E-03 | 1.20E-03 | 2.89E-05 | 4.04E-02 | 9.39E-04 | 1.45E-04 | 1.30E-04 | 8.04E-04 | 5.25E-04 |  | 8.20E-06 | 8.02E-03 |
| **K2** | 1.09E-02 | 2.18E-03 | 9.59E-04 | 2.73E-05 | 4.16E-02 | 1.03E-03 | 5.58E-05 | 1.06E-04 | 6.05E-04 | 1.99E-04 |  | 9.55E-06 | 2.90E-03 |
| **K3** | 1.22E-02 | 2.73E-03 | 9.96E-04 | 2.70E-05 | 3.85E-02 | 9.60E-04 | 5.07E-05 | 1.09E-04 | 5.64E-04 | 4.74E-05 |  | 4.62E-06 | 3.35E-03 |

Table S5. The Chronic Daily Intake (CDI) for dermal absorption in females.

| **Label** | **Al** | **Ti** | **Cr** | **Mn** | **Fe** | **Co** | **Ni** | **Cu** | **Zn** | **Cd** | **Hg** | **Pb** |
| --- | --- | --- | --- | --- | --- | --- | --- | --- | --- | --- | --- | --- |
| **A2** | 1.27E-02 | 1.64E-02 | 1.88E-03 | 6.92E-05 | 1.13E-02 | 2.09E-04 | 2.11E-05 | 1.63E-04 | 7.12E-04 | 1.80E-05 | 6.66E-05 | 3.34E-03 |
| **A3** | 1.34E-02 | 1.24E-02 | 1.47E-03 | 5.99E-05 | 1.71E-02 | 2.26E-04 | 1.10E-04 | 2.95E-04 | 1.12E-03 | 2.37E-05 | 5.97E-06 | 7.75E-03 |
| **B1** | 1.06E-02 | 2.85E-03 | 1.46E-03 | 3.87E-05 | 2.87E-02 | 8.63E-04 | 8.05E-05 | 2.01E-04 | 7.69E-04 | 2.69E-05 | 1.09E-05 | 6.17E-03 |
| **B2** | 9.88E-03 | 4.03E-03 | 1.30E-03 | 2.93E-05 | 3.62E-02 | 8.13E-04 | 3.83E-05 | 1.66E-04 | 9.17E-04 | 6.87E-05 | 1.90E-05 | 5.55E-03 |
| **B3** | 1.08E-02 | 4.00E-03 | 1.24E-03 | 5.87E-05 | 3.10E-02 | 8.42E-04 | 4.11E-05 | 1.21E-04 | 1.06E-03 | 1.06E-02 | 3.58E-06 | 3.20E-03 |
| **C1** | 1.17E-02 | 2.70E-03 | 8.92E-04 | 3.29E-05 | 4.28E-02 | 9.48E-04 | 4.35E-05 | 1.58E-04 | 1.65E-03 | 9.88E-05 | 1.11E-05 | 3.70E-03 |
| **C2** | 6.39E-03 | 2.49E-03 | 9.60E-04 | 3.39E-05 | 1.99E-02 | 6.18E-04 | 4.03E-05 | 9.57E-05 | 7.11E-04 | 4.82E-04 | 1.92E-05 | 4.04E-03 |
| **C3** | 8.18E-03 | 2.66E-03 | 9.85E-04 | 2.57E-05 | 2.80E-02 | 8.20E-04 | 4.91E-05 | 1.16E-04 | 7.43E-04 | 3.19E-05 | 2.65E-06 | 3.85E-03 |
| **D1** | 8.28E-03 | 1.80E-03 | 1.12E-03 | 2.90E-05 | 5.25E-02 | 1.53E-03 | 4.37E-05 | 1.43E-04 | 7.33E-04 | 2.32E-05 | 2.92E-06 | 2.43E-03 |
| **D2** | 1.10E-02 | 2.54E-03 | 3.36E-04 | 2.51E-05 | 4.72E-02 | 6.84E-04 | 4.35E-05 | 1.14E-04 | 7.77E-04 | 3.99E-05 | 3.58E-06 | 3.13E-03 |
| **E1** | 4.41E-03 | 1.42E-03 | 1.00E-03 | 1.71E-05 | 2.57E-02 | 4.00E-04 | 3.05E-05 | 6.84E-05 | 4.64E-04 | 5.79E-05 | 2.92E-06 | 3.47E-03 |
| **E2** | 1.11E-02 | 2.50E-03 | 8.90E-04 | 2.29E-05 | 3.09E-02 | 7.92E-04 | 3.23E-05 | 1.09E-04 | 6.32E-04 | 6.51E-04 | 3.71E-06 | 2.19E-03 |
| **E3** | 5.77E-03 | 2.12E-03 | 4.10E-04 | 1.25E-05 | 1.23E-02 | 2.93E-04 | 1.91E-05 | 3.79E-05 | 2.66E-04 | 6.23E-05 | 1.33E-06 | 1.74E-03 |
| **F1** | 7.58E-03 | 2.18E-03 | 1.12E-03 | 1.86E-05 | 2.50E-02 | 6.22E-04 | 3.09E-05 | 9.98E-05 | 5.52E-04 | 2.90E-05 | 6.89E-06 | 2.83E-03 |
| **F2** | 1.42E-02 | 3.85E-03 | 1.77E-03 | 3.16E-05 | 4.94E-02 | 1.40E-03 | 5.01E-05 | 1.31E-04 | 5.39E-04 | 2.43E-05 | 5.17E-06 | 1.69E-03 |
| **F3** | 9.94E-03 | 6.24E-03 | 1.17E-03 | 2.49E-05 | 3.26E-02 | 8.37E-04 | 2.44E-05 | 1.05E-04 | 6.33E-04 | 2.49E-05 | 6.76E-06 | 1.58E-03 |
| **G1** | 6.51E-03 | 1.98E-03 | 6.91E-04 | 1.39E-05 | 2.38E-02 | 4.19E-04 | 2.52E-05 | 7.66E-05 | 4.41E-04 | 2.02E-05 | 2.12E-06 | 1.48E-03 |
| **G2** | 1.30E-02 | 2.23E-03 | 1.16E-03 | 2.84E-05 | 4.13E-02 | 1.12E-03 | 3.29E-05 | 1.31E-04 | 5.72E-04 | 3.18E-05 | 4.30E-05 | 1.18E-03 |
| **G3** | 9.55E-03 | 2.34E-03 | 8.76E-04 | 2.35E-05 | 3.15E-02 | 8.69E-04 | 2.62E-05 | 1.03E-04 | 6.31E-04 | 3.92E-05 | 3.58E-06 | 1.80E-03 |
| **H1** | 3.34E-03 | 8.80E-04 | 2.94E-04 | 9.94E-06 | 9.94E-03 | 2.24E-04 | 1.13E-05 | 5.59E-05 | 3.43E-04 | 3.01E-05 | 1.06E-06 | 6.81E-04 |
| **H2** | 6.32E-03 | 1.34E-03 | 6.66E-04 | 7.09E-05 | 2.01E-02 | 5.05E-04 | 3.39E-05 | 1.03E-04 | 9.51E-04 | 8.96E-03 | 3.41E-05 | 7.40E-03 |
| **H3** | 5.63E-03 | 3.17E-03 | 5.60E-04 | 1.71E-05 | 1.20E-02 | 3.00E-04 | 1.18E-05 | 4.11E-05 | 3.00E-04 | 7.98E-04 | 1.38E-05 | 1.70E-03 |
| **I1** | 9.71E-03 | 2.02E-03 | 1.08E-03 | 2.36E-05 | 3.35E-02 | 8.55E-04 | 3.05E-05 | 9.51E-05 | 4.41E-04 | 2.09E-04 | 3.71E-06 | 1.43E-03 |
| **I2** | 9.35E-03 | 1.53E-03 | 7.83E-04 | 2.60E-05 | 3.86E-02 | 9.45E-04 | 3.50E-05 | 1.04E-04 | 5.48E-04 | 1.42E-04 | 2.92E-06 | 1.80E-03 |
| **I3** | 5.54E-03 | 2.98E-03 | 5.61E-04 | 1.55E-05 | 1.63E-02 | 2.89E-04 | 2.49E-05 | 4.28E-05 | 2.91E-04 | 7.97E-05 | 2.13E-05 | 3.02E-03 |
| **J1** | 4.24E-03 | 1.44E-03 | 4.45E-04 | 1.60E-05 | 1.70E-02 | 4.08E-04 | 7.62E-05 | 5.69E-05 | 3.23E-04 | 1.11E-04 | 3.09E-05 | 5.78E-03 |
| **J2** | 3.63E-03 | 2.12E-03 | 3.33E-04 | 1.71E-05 | 1.52E-02 | 2.26E-04 | 9.23E-05 | 6.18E-05 | 4.02E-04 | 1.54E-04 | 6.36E-06 | 4.84E-03 |
| **J3** | 6.27E-03 | 2.59E-03 | 5.54E-04 | 1.87E-05 | 1.82E-02 | 5.56E-04 | 3.99E-05 | 6.71E-05 | 3.04E-04 | 1.12E-04 | 3.84E-06 | 3.88E-03 |
| **K1** | 1.10E-02 | 2.49E-03 | 1.07E-03 | 2.57E-05 | 3.59E-02 | 8.35E-04 | 1.29E-04 | 1.15E-04 | 7.15E-04 | 4.66E-04 | 7.29E-06 | 7.13E-03 |
| **K2** | 9.70E-03 | 1.94E-03 | 8.52E-04 | 2.43E-05 | 3.70E-02 | 9.13E-04 | 4.96E-05 | 9.41E-05 | 5.37E-04 | 1.77E-04 | 8.48E-06 | 2.58E-03 |
| **K3** | 1.09E-02 | 2.43E-03 | 8.85E-04 | 2.40E-05 | 3.42E-02 | 8.53E-04 | 4.51E-05 | 9.73E-05 | 5.02E-04 | 4.22E-05 | 4.11E-06 | 2.97E-03 |

Table S6. The Chronic Daily Intake (CDI) for dermal absorption in children.

| **Label** | **Al** | **Ti** | **Cr** | **Mn** | **Fe** | **Co** | **Ni** | **Cu** | **Zn** | **Cd** | **Hg** | **Pb** |
| --- | --- | --- | --- | --- | --- | --- | --- | --- | --- | --- | --- | --- |
| **A2** | 4.68E-02 | 6.07E-02 | 6.95E-03 | 2.56E-04 | 4.17E-02 | 7.74E-04 | 7.79E-05 | 6.04E-04 | 2.63E-03 | 6.66E-05 | 2.46E-04 | 1.23E-02 |
| **A3** | 4.94E-02 | 4.59E-02 | 5.42E-03 | 2.21E-04 | 6.33E-02 | 8.35E-04 | 4.05E-04 | 1.09E-03 | 4.12E-03 | 8.77E-05 | 2.21E-05 | 2.86E-02 |
| **B1** | 3.92E-02 | 1.05E-02 | 5.40E-03 | 1.43E-04 | 1.06E-01 | 3.19E-03 | 2.97E-04 | 7.42E-04 | 2.84E-03 | 9.95E-05 | 4.02E-05 | 2.28E-02 |
| **B2** | 3.65E-02 | 1.49E-02 | 4.81E-03 | 1.08E-04 | 1.34E-01 | 3.01E-03 | 1.42E-04 | 6.14E-04 | 3.39E-03 | 2.54E-04 | 7.01E-05 | 2.05E-02 |
| **B3** | 3.99E-02 | 1.48E-02 | 4.60E-03 | 2.17E-04 | 1.15E-01 | 3.11E-03 | 1.52E-04 | 4.46E-04 | 3.91E-03 | 3.91E-02 | 1.32E-05 | 1.18E-02 |
| **C1** | 4.31E-02 | 9.99E-03 | 3.30E-03 | 1.22E-04 | 1.58E-01 | 3.50E-03 | 1.61E-04 | 5.86E-04 | 6.10E-03 | 3.65E-04 | 4.12E-05 | 1.37E-02 |
| **C2** | 2.36E-02 | 9.20E-03 | 3.55E-03 | 1.25E-04 | 7.35E-02 | 2.28E-03 | 1.49E-04 | 3.54E-04 | 2.63E-03 | 1.78E-03 | 7.11E-05 | 1.49E-02 |
| **C3** | 3.02E-02 | 9.83E-03 | 3.64E-03 | 9.51E-05 | 1.04E-01 | 3.03E-03 | 1.81E-04 | 4.29E-04 | 2.75E-03 | 1.18E-04 | 9.80E-06 | 1.42E-02 |
| **D1** | 3.06E-02 | 6.64E-03 | 4.12E-03 | 1.07E-04 | 1.94E-01 | 5.66E-03 | 1.62E-04 | 5.28E-04 | 2.71E-03 | 8.58E-05 | 1.08E-05 | 8.98E-03 |
| **D2** | 4.07E-02 | 9.38E-03 | 1.24E-03 | 9.26E-05 | 1.75E-01 | 2.53E-03 | 1.61E-04 | 4.22E-04 | 2.87E-03 | 1.47E-04 | 1.32E-05 | 1.16E-02 |
| **E1** | 1.63E-02 | 5.25E-03 | 3.70E-03 | 6.32E-05 | 9.49E-02 | 1.48E-03 | 1.13E-04 | 2.53E-04 | 1.71E-03 | 2.14E-04 | 1.08E-05 | 1.28E-02 |
| **E2** | 4.09E-02 | 9.23E-03 | 3.29E-03 | 8.48E-05 | 1.14E-01 | 2.93E-03 | 1.20E-04 | 4.03E-04 | 2.33E-03 | 2.40E-03 | 1.37E-05 | 8.09E-03 |
| **E3** | 2.13E-02 | 7.82E-03 | 1.52E-03 | 4.61E-05 | 4.54E-02 | 1.08E-03 | 7.06E-05 | 1.40E-04 | 9.82E-04 | 2.30E-04 | 4.90E-06 | 6.43E-03 |
| **F1** | 2.80E-02 | 8.05E-03 | 4.16E-03 | 6.86E-05 | 9.22E-02 | 2.30E-03 | 1.14E-04 | 3.69E-04 | 2.04E-03 | 1.07E-04 | 2.55E-05 | 1.05E-02 |
| **F2** | 5.24E-02 | 1.42E-02 | 6.56E-03 | 1.17E-04 | 1.82E-01 | 5.17E-03 | 1.85E-04 | 4.86E-04 | 1.99E-03 | 8.97E-05 | 1.91E-05 | 6.25E-03 |
| **F3** | 3.67E-02 | 2.31E-02 | 4.32E-03 | 9.21E-05 | 1.21E-01 | 3.09E-03 | 9.02E-05 | 3.89E-04 | 2.34E-03 | 9.21E-05 | 2.50E-05 | 5.83E-03 |
| **G1** | 2.41E-02 | 7.33E-03 | 2.55E-03 | 5.15E-05 | 8.78E-02 | 1.55E-03 | 9.31E-05 | 2.83E-04 | 1.63E-03 | 7.45E-05 | 7.84E-06 | 5.49E-03 |
| **G2** | 4.81E-02 | 8.25E-03 | 4.30E-03 | 1.05E-04 | 1.53E-01 | 4.14E-03 | 1.22E-04 | 4.85E-04 | 2.11E-03 | 1.18E-04 | 1.59E-04 | 4.38E-03 |
| **G3** | 3.53E-02 | 8.64E-03 | 3.24E-03 | 8.67E-05 | 1.17E-01 | 3.21E-03 | 9.70E-05 | 3.80E-04 | 2.33E-03 | 1.45E-04 | 1.32E-05 | 6.66E-03 |
| **H1** | 1.23E-02 | 3.25E-03 | 1.09E-03 | 3.68E-05 | 3.67E-02 | 8.28E-04 | 4.17E-05 | 2.07E-04 | 1.27E-03 | 1.11E-04 | 3.92E-06 | 2.52E-03 |
| **H2** | 2.33E-02 | 4.94E-03 | 2.46E-03 | 2.62E-04 | 7.43E-02 | 1.87E-03 | 1.25E-04 | 3.82E-04 | 3.51E-03 | 3.31E-02 | 1.26E-04 | 2.73E-02 |
| **H3** | 2.08E-02 | 1.17E-02 | 2.07E-03 | 6.32E-05 | 4.45E-02 | 1.11E-03 | 4.36E-05 | 1.52E-04 | 1.11E-03 | 2.95E-03 | 5.10E-05 | 6.27E-03 |
| **I1** | 3.59E-02 | 7.47E-03 | 4.00E-03 | 8.72E-05 | 1.24E-01 | 3.16E-03 | 1.13E-04 | 3.51E-04 | 1.63E-03 | 7.74E-04 | 1.37E-05 | 5.29E-03 |
| **I2** | 3.45E-02 | 5.67E-03 | 2.89E-03 | 9.60E-05 | 1.43E-01 | 3.49E-03 | 1.29E-04 | 3.83E-04 | 2.02E-03 | 5.26E-04 | 1.08E-05 | 6.66E-03 |
| **I3** | 2.05E-02 | 1.10E-02 | 2.07E-03 | 5.73E-05 | 6.01E-02 | 1.07E-03 | 9.21E-05 | 1.58E-04 | 1.07E-03 | 2.94E-04 | 7.89E-05 | 1.11E-02 |
| **J1** | 1.57E-02 | 5.33E-03 | 1.65E-03 | 5.93E-05 | 6.29E-02 | 1.51E-03 | 2.82E-04 | 2.10E-04 | 1.19E-03 | 4.11E-04 | 1.14E-04 | 2.14E-02 |
| **J2** | 1.34E-02 | 7.83E-03 | 1.23E-03 | 6.32E-05 | 5.63E-02 | 8.33E-04 | 3.41E-04 | 2.28E-04 | 1.49E-03 | 5.69E-04 | 2.35E-05 | 1.79E-02 |
| **J3** | 2.32E-02 | 9.58E-03 | 2.05E-03 | 6.91E-05 | 6.72E-02 | 2.05E-03 | 1.47E-04 | 2.48E-04 | 1.12E-03 | 4.13E-04 | 1.42E-05 | 1.43E-02 |
| **K1** | 4.07E-02 | 9.20E-03 | 3.94E-03 | 9.51E-05 | 1.33E-01 | 3.09E-03 | 4.78E-04 | 4.26E-04 | 2.64E-03 | 1.72E-03 | 2.70E-05 | 2.64E-02 |
| **K2** | 3.59E-02 | 7.18E-03 | 3.15E-03 | 8.97E-05 | 1.37E-01 | 3.38E-03 | 1.83E-04 | 3.48E-04 | 1.99E-03 | 6.53E-04 | 3.14E-05 | 9.54E-03 |
| **K3** | 4.02E-02 | 8.97E-03 | 3.27E-03 | 8.87E-05 | 1.26E-01 | 3.15E-03 | 1.67E-04 | 3.60E-04 | 1.85E-03 | 1.56E-04 | 1.52E-05 | 1.10E-02 |

Table S7. The values of HQ for males, females, and children.

| **Label** | **Males** | | | | | | **Females** | | | | | | **Children** | | | | | |
| --- | --- | --- | --- | --- | --- | --- | --- | --- | --- | --- | --- | --- | --- | --- | --- | --- | --- | --- |
|  | **Cd** | **Cu** | **Fe** | **Mn** | **Pb** | **Zn** | **Cd** | **Cu** | **Fe** | **Mn** | **Pb** | **Zn** | **Cd** | **Cu** | **Fe** | **Mn** | **Pb** | **Zn** |
| **A2** | 0.0203 | 0.0046 | 0.0159 | 0.0006 | 1.0682 | 0.0027 | 0.0180 | 0.0041 | 0.0141 | 0.0005 | 0.9489 | 0.0024 | 0.0666 | 0.0151 | 0.0521 | 0.0018 | 3.4943 | 0.0088 |
| **A3** | 0.0267 | 0.0083 | 0.0241 | 0.0005 | 2.4773 | 0.0042 | 0.0237 | 0.0074 | 0.0214 | 0.0004 | 2.2017 | 0.0037 | 0.0877 | 0.0273 | 0.0791 | 0.0016 | 8.1250 | 0.0137 |
| **B1** | 0.0303 | 0.0057 | 0.0404 | 0.0003 | 1.9716 | 0.0029 | 0.0269 | 0.0050 | 0.0359 | 0.0003 | 1.7528 | 0.0026 | 0.0995 | 0.0186 | 0.1325 | 0.0010 | 6.4773 | 0.0095 |
| **B2** | 0.0773 | 0.0047 | 0.0510 | 0.0002 | 1.7756 | 0.0034 | 0.0687 | 0.0042 | 0.0453 | 0.0002 | 1.5767 | 0.0031 | 0.2540 | 0.0154 | 0.1675 | 0.0008 | 5.8239 | 0.0113 |
| **B3** | 11.9000 | 0.0034 | 0.0436 | 0.0005 | 1.0227 | 0.0040 | 10.6000 | 0.0030 | 0.0388 | 0.0004 | 0.9091 | 0.0035 | 39.1000 | 0.0112 | 0.1438 | 0.0016 | 3.3523 | 0.0130 |
| **C1** | 0.1110 | 0.0045 | 0.0603 | 0.0003 | 1.1818 | 0.0062 | 0.0988 | 0.0040 | 0.0535 | 0.0002 | 1.0511 | 0.0055 | 0.3650 | 0.0147 | 0.1975 | 0.0009 | 3.8920 | 0.0203 |
| **C2** | 0.5430 | 0.0027 | 0.0280 | 0.0003 | 1.2898 | 0.0027 | 0.4820 | 0.0024 | 0.0249 | 0.0002 | 1.1477 | 0.0024 | 1.7800 | 0.0089 | 0.0919 | 0.0009 | 4.2330 | 0.0088 |
| **C3** | 0.0359 | 0.0033 | 0.0394 | 0.0002 | 1.2301 | 0.0028 | 0.0319 | 0.0029 | 0.0350 | 0.0002 | 1.0938 | 0.0025 | 0.1180 | 0.0107 | 0.1300 | 0.0007 | 4.0341 | 0.0092 |
| **D1** | 0.0261 | 0.0040 | 0.0738 | 0.0002 | 0.7756 | 0.0028 | 0.0232 | 0.0036 | 0.0656 | 0.0002 | 0.6903 | 0.0024 | 0.0858 | 0.0132 | 0.2425 | 0.0008 | 2.5511 | 0.0090 |
| **D2** | 0.0449 | 0.0032 | 0.0664 | 0.0002 | 1.0000 | 0.0029 | 0.0399 | 0.0029 | 0.0590 | 0.0002 | 0.8892 | 0.0026 | 0.1470 | 0.0106 | 0.2188 | 0.0007 | 3.2955 | 0.0096 |
| **E1** | 0.0652 | 0.0019 | 0.0361 | 0.0001 | 1.1108 | 0.0017 | 0.0579 | 0.0017 | 0.0321 | 0.0001 | 0.9858 | 0.0015 | 0.2140 | 0.0063 | 0.1186 | 0.0005 | 3.6364 | 0.0057 |
| **E2** | 0.7320 | 0.0031 | 0.0435 | 0.0002 | 0.6989 | 0.0024 | 0.6510 | 0.0027 | 0.0386 | 0.0002 | 0.6222 | 0.0021 | 2.4000 | 0.0101 | 0.1425 | 0.0006 | 2.2983 | 0.0078 |
| **E3** | 0.0701 | 0.0011 | 0.0173 | 0.0001 | 0.5568 | 0.0010 | 0.0623 | 0.0009 | 0.0154 | 0.0001 | 0.4943 | 0.0009 | 0.2300 | 0.0035 | 0.0568 | 0.0003 | 1.8267 | 0.0033 |
| **F1** | 0.0327 | 0.0028 | 0.0351 | 0.0001 | 0.9034 | 0.0021 | 0.0290 | 0.0025 | 0.0313 | 0.0001 | 0.8040 | 0.0018 | 0.1070 | 0.0092 | 0.1153 | 0.0005 | 2.9830 | 0.0068 |
| **F2** | 0.0273 | 0.0037 | 0.0694 | 0.0003 | 0.5398 | 0.0020 | 0.0243 | 0.0033 | 0.0618 | 0.0002 | 0.4801 | 0.0018 | 0.0897 | 0.0122 | 0.2275 | 0.0008 | 1.7756 | 0.0066 |
| **F3** | 0.0280 | 0.0030 | 0.0459 | 0.0002 | 0.5057 | 0.0024 | 0.0249 | 0.0026 | 0.0408 | 0.0002 | 0.4489 | 0.0021 | 0.0921 | 0.0097 | 0.1513 | 0.0007 | 1.6563 | 0.0078 |
| **G1** | 0.0227 | 0.0022 | 0.0334 | 0.0001 | 0.4744 | 0.0017 | 0.0202 | 0.0019 | 0.0298 | 0.0001 | 0.4205 | 0.0015 | 0.0745 | 0.0071 | 0.1098 | 0.0004 | 1.5597 | 0.0054 |
| **G2** | 0.0358 | 0.0037 | 0.0581 | 0.0002 | 0.3778 | 0.0021 | 0.0318 | 0.0033 | 0.0516 | 0.0002 | 0.3352 | 0.0019 | 0.1180 | 0.0121 | 0.1913 | 0.0008 | 1.2443 | 0.0070 |
| **G3** | 0.0441 | 0.0029 | 0.0444 | 0.0002 | 0.5767 | 0.0024 | 0.0392 | 0.0026 | 0.0394 | 0.0002 | 0.5114 | 0.0021 | 0.1450 | 0.0095 | 0.1463 | 0.0006 | 1.8920 | 0.0078 |
| **H1** | 0.0339 | 0.0016 | 0.0140 | 0.0001 | 0.2176 | 0.0013 | 0.0301 | 0.0014 | 0.0124 | 0.0001 | 0.1935 | 0.0011 | 0.1110 | 0.0052 | 0.0459 | 0.0003 | 0.7159 | 0.0042 |
| **H2** | 10.1000 | 0.0029 | 0.0283 | 0.0006 | 2.3636 | 0.0036 | 8.9600 | 0.0026 | 0.0251 | 0.0005 | 2.1023 | 0.0032 | 33.1000 | 0.0096 | 0.0929 | 0.0019 | 7.7557 | 0.0117 |
| **H3** | 0.8980 | 0.0012 | 0.0170 | 0.0001 | 0.5426 | 0.0011 | 0.7980 | 0.0010 | 0.0150 | 0.0001 | 0.4830 | 0.0010 | 2.9500 | 0.0038 | 0.0556 | 0.0005 | 1.7813 | 0.0037 |
| **I1** | 0.2350 | 0.0027 | 0.0471 | 0.0002 | 0.4574 | 0.0017 | 0.2090 | 0.0024 | 0.0419 | 0.0002 | 0.4063 | 0.0015 | 0.7740 | 0.0088 | 0.1550 | 0.0006 | 1.5028 | 0.0054 |
| **I2** | 0.1600 | 0.0029 | 0.0543 | 0.0002 | 0.5767 | 0.0021 | 0.1420 | 0.0026 | 0.0483 | 0.0002 | 0.5114 | 0.0018 | 0.5260 | 0.0096 | 0.1788 | 0.0007 | 1.8920 | 0.0067 |
| **I3** | 0.0896 | 0.0012 | 0.0229 | 0.0001 | 0.9631 | 0.0011 | 0.0797 | 0.0011 | 0.0204 | 0.0001 | 0.8580 | 0.0010 | 0.2940 | 0.0040 | 0.0751 | 0.0004 | 3.1534 | 0.0036 |
| **J1** | 0.1250 | 0.0016 | 0.0239 | 0.0001 | 1.8466 | 0.0012 | 0.1110 | 0.0014 | 0.0213 | 0.0001 | 1.6420 | 0.0011 | 0.4110 | 0.0053 | 0.0786 | 0.0004 | 6.0795 | 0.0040 |
| **J2** | 0.1730 | 0.0017 | 0.0214 | 0.0001 | 1.5483 | 0.0015 | 0.1540 | 0.0015 | 0.0190 | 0.0001 | 1.3750 | 0.0013 | 0.5690 | 0.0057 | 0.0704 | 0.0005 | 5.0852 | 0.0050 |
| **J3** | 0.1260 | 0.0019 | 0.0256 | 0.0002 | 1.2415 | 0.0011 | 0.1120 | 0.0017 | 0.0228 | 0.0001 | 1.1023 | 0.0010 | 0.4130 | 0.0062 | 0.0840 | 0.0005 | 4.0625 | 0.0037 |
| **K1** | 0.5250 | 0.0033 | 0.0505 | 0.0002 | 2.2784 | 0.0027 | 0.4660 | 0.0029 | 0.0449 | 0.0002 | 2.0256 | 0.0024 | 1.7200 | 0.0107 | 0.1663 | 0.0007 | 7.5000 | 0.0088 |
| **K2** | 0.1990 | 0.0027 | 0.0520 | 0.0002 | 0.8239 | 0.0020 | 0.1770 | 0.0024 | 0.0463 | 0.0002 | 0.7330 | 0.0018 | 0.6530 | 0.0087 | 0.1713 | 0.0006 | 2.7102 | 0.0066 |
| **K3** | 0.0474 | 0.0027 | 0.0481 | 0.0002 | 0.9517 | 0.0019 | 0.0422 | 0.0024 | 0.0428 | 0.0002 | 0.8438 | 0.0017 | 0.1560 | 0.0090 | 0.1575 | 0.0006 | 3.1250 | 0.0062 |

Table S8. The values of HI for males, females, and children.

| **Label** | **Hazard Index (HI)** | | |
| --- | --- | --- | --- |
|  | **Males** | **Females** | **Children** |
| **A2** | 1.11E+00 | 9.88E-01 | 3.64E+00 |
| **A3** | 2.54E+00 | 2.26E+00 | 8.33E+00 |
| **B1** | 2.05E+00 | 1.82E+00 | 6.74E+00 |
| **B2** | 1.91E+00 | 1.70E+00 | 6.27E+00 |
| **B3** | 1.30E+01 | 1.16E+01 | 4.26E+01 |
| **C1** | 1.36E+00 | 1.21E+00 | 4.49E+00 |
| **C2** | 1.87E+00 | 1.66E+00 | 6.12E+00 |
| **C3** | 1.31E+00 | 1.17E+00 | 4.30E+00 |
| **D1** | 8.82E-01 | 7.85E-01 | 2.90E+00 |
| **D2** | 1.12E+00 | 9.94E-01 | 3.68E+00 |
| **E1** | 1.22E+00 | 1.08E+00 | 3.98E+00 |
| **E2** | 1.48E+00 | 1.32E+00 | 4.86E+00 |
| **E3** | 6.46E-01 | 5.74E-01 | 2.12E+00 |
| **F1** | 9.76E-01 | 8.69E-01 | 3.22E+00 |
| **F2** | 6.42E-01 | 5.71E-01 | 2.11E+00 |
| **F3** | 5.85E-01 | 5.19E-01 | 1.92E+00 |
| **G1** | 5.34E-01 | 4.74E-01 | 1.76E+00 |
| **G2** | 4.78E-01 | 4.24E-01 | 1.57E+00 |
| **G3** | 6.71E-01 | 5.95E-01 | 2.20E+00 |
| **H1** | 2.68E-01 | 2.39E-01 | 8.82E-01 |
| **H2** | 1.25E+01 | 1.11E+01 | 4.10E+01 |
| **H3** | 1.46E+00 | 1.30E+00 | 4.79E+00 |
| **I1** | 7.44E-01 | 6.61E-01 | 2.45E+00 |
| **I2** | 7.96E-01 | 7.06E-01 | 2.61E+00 |
| **I3** | 1.08E+00 | 9.60E-01 | 3.53E+00 |
| **J1** | 2.00E+00 | 1.78E+00 | 6.58E+00 |
| **J2** | 1.75E+00 | 1.55E+00 | 5.74E+00 |
| **J3** | 1.40E+00 | 1.24E+00 | 4.57E+00 |
| **K1** | 2.86E+00 | 2.54E+00 | 9.41E+00 |
| **K2** | 1.08E+00 | 9.61E-01 | 3.55E+00 |
| **K3** | 1.05E+00 | 9.33E-01 | 3.45E+00 |

Table S9. The values of *CR* for males, females, and children.

| **Label** | **Males** | | **Females** | | **Children** | |
| --- | --- | --- | --- | --- | --- | --- |
|  | **Cd** | **Pb** | **Cd** | **Pb** | **Cd** | **Pb** |
| **A2** | 3.05E-04 | 3.20E-05 | 2.70E-04 | 2.84E-05 | 9.99E-04 | 1.05E-04 |
| **A3** | 4.01E-04 | 7.41E-05 | 3.56E-04 | 6.59E-05 | 1.32E-03 | 2.43E-04 |
| **B1** | 4.55E-04 | 5.90E-05 | 4.04E-04 | 5.24E-05 | 1.49E-03 | 1.94E-04 |
| **B2** | 1.16E-03 | 5.31E-05 | 1.03E-03 | 4.72E-05 | 3.81E-03 | 1.74E-04 |
| **B3** | 1.79E-01 | 3.06E-05 | 1.59E-01 | 2.72E-05 | 5.87E-01 | 1.00E-04 |
| **C1** | 1.67E-03 | 3.54E-05 | 1.48E-03 | 3.15E-05 | 5.48E-03 | 1.16E-04 |
| **C2** | 8.15E-03 | 3.86E-05 | 7.23E-03 | 3.43E-05 | 2.67E-02 | 1.27E-04 |
| **C3** | 5.39E-04 | 3.68E-05 | 4.79E-04 | 3.27E-05 | 1.77E-03 | 1.21E-04 |
| **D1** | 3.92E-04 | 2.32E-05 | 3.48E-04 | 2.07E-05 | 1.29E-03 | 7.63E-05 |
| **D2** | 6.74E-04 | 2.99E-05 | 5.99E-04 | 2.66E-05 | 2.21E-03 | 9.86E-05 |
| **E1** | 9.78E-04 | 3.32E-05 | 8.69E-04 | 2.95E-05 | 3.21E-03 | 1.09E-04 |
| **E2** | 1.10E-02 | 2.09E-05 | 9.77E-03 | 1.86E-05 | 3.60E-02 | 6.88E-05 |
| **E3** | 1.05E-03 | 1.67E-05 | 9.35E-04 | 1.48E-05 | 3.45E-03 | 5.47E-05 |
| **F1** | 4.91E-04 | 2.70E-05 | 4.35E-04 | 2.41E-05 | 1.61E-03 | 8.93E-05 |
| **F2** | 4.10E-04 | 1.62E-05 | 3.65E-04 | 1.44E-05 | 1.35E-03 | 5.31E-05 |
| **F3** | 4.20E-04 | 1.51E-05 | 3.74E-04 | 1.34E-05 | 1.38E-03 | 4.96E-05 |
| **G1** | 3.41E-04 | 1.42E-05 | 3.03E-04 | 1.26E-05 | 1.12E-03 | 4.67E-05 |
| **G2** | 5.37E-04 | 1.13E-05 | 4.77E-04 | 1.00E-05 | 1.77E-03 | 3.72E-05 |
| **G3** | 6.62E-04 | 1.73E-05 | 5.88E-04 | 1.53E-05 | 2.18E-03 | 5.66E-05 |
| **H1** | 5.09E-04 | 6.51E-06 | 4.52E-04 | 5.79E-06 | 1.67E-03 | 2.14E-05 |
| **H2** | 1.52E-01 | 7.07E-05 | 1.34E-01 | 6.29E-05 | 4.97E-01 | 2.32E-04 |
| **H3** | 1.35E-02 | 1.62E-05 | 1.20E-02 | 1.45E-05 | 4.43E-02 | 5.33E-05 |
| **I1** | 3.53E-03 | 1.37E-05 | 3.14E-03 | 1.22E-05 | 1.16E-02 | 4.50E-05 |
| **I2** | 2.40E-03 | 1.73E-05 | 2.13E-03 | 1.53E-05 | 7.89E-03 | 5.66E-05 |
| **I3** | 1.34E-03 | 2.88E-05 | 1.20E-03 | 2.57E-05 | 4.41E-03 | 9.44E-05 |
| **J1** | 1.88E-03 | 5.53E-05 | 1.67E-03 | 4.91E-05 | 6.17E-03 | 1.82E-04 |
| **J2** | 2.60E-03 | 4.63E-05 | 2.31E-03 | 4.11E-05 | 8.54E-03 | 1.52E-04 |
| **J3** | 1.89E-03 | 3.71E-05 | 1.68E-03 | 3.30E-05 | 6.20E-03 | 1.22E-04 |
| **K1** | 7.88E-03 | 6.82E-05 | 6.99E-03 | 6.06E-05 | 2.58E-02 | 2.24E-04 |
| **K2** | 2.99E-03 | 2.47E-05 | 2.66E-03 | 2.19E-05 | 9.80E-03 | 8.11E-05 |
| **K3** | 7.11E-04 | 2.85E-05 | 6.33E-04 | 2.52E-05 | 2.34E-03 | 9.35E-05 |

Table S10. Pearson’s correlation matrix.

|  | **_Li_** | **_B_** | **_Na_** | **_Mg_** | **_Al_** | **_K_** | **_Ca_** | **_Ti_** | **_Cr_** | **_Mn_** | **_Fe_** | **_Co_** | **_Ni_** | **_Cu_** | **_Zn_** | **_Ga_** | **_Se_** | **_Sr_** | **_Ag_** | **_Cd_** | **_In_** | **_Ba_** | **_Hg_** | **_Pb_** | **_Bi_** |
| --- | --- | --- | --- | --- | --- | --- | --- | --- | --- | --- | --- | --- | --- | --- | --- | --- | --- | --- | --- | --- | --- | --- | --- | --- | --- |
| **_Li_** | _1_ |  |  |  |  |  |  |  |  |  |  |  |  |  |  |  |  |  |  |  |  |  |  |  |  |
| **_B_** | _-0.154_ | _1_ |  |  |  |  |  |  |  |  |  |  |  |  |  |  |  |  |  |  |  |  |  |  |  |
| **_Na_** | _-0.023_ | **_.736**_** | _1_ |  |  |  |  |  |  |  |  |  |  |  |  |  |  |  |  |  |  |  |  |  |  |
| **_Mg_** | _-0.014_ | _.661**_ | _.553**_ | _1_ |  |  |  |  |  |  |  |  |  |  |  |  |  |  |  |  |  |  |  |  |  |
| **_Al_** | _.679**_ | _.386*_ | _.403*_ | _.391*_ | _1_ |  |  |  |  |  |  |  |  |  |  |  |  |  |  |  |  |  |  |  |  |
| **_K_** | _.889**_ | _-0.304_ | _-0.012_ | _-0.186_ | _.676**_ | _1_ |  |  |  |  |  |  |  |  |  |  |  |  |  |  |  |  |  |  |  |
| **_Ca_** | _0.14_ | _.655**_ | _.741**_ | **_.871**_** | _.538**_ | _0.074_ | _1_ |  |  |  |  |  |  |  |  |  |  |  |  |  |  |  |  |  |  |
| **_Ti_** | _-0.049_ | **_.900**_** | _.571**_ | _.665**_ | _.467**_ | _-0.241_ | _.534**_ | _1_ |  |  |  |  |  |  |  |  |  |  |  |  |  |  |  |  |  |
| **_Cr_** | _.379*_ | _.613**_ | _.558**_ | _.397*_ | **_.723**_** | _0.33_ | _.538**_ | _.613**_ | _1_ |  |  |  |  |  |  |  |  |  |  |  |  |  |  |  |  |
| **_Mn_** | _0.347_ | _.648**_ | _.543**_ | _.484**_ | _.482**_ | _0.072_ | _.519**_ | _.627**_ | _.580**_ | _1_ |  |  |  |  |  |  |  |  |  |  |  |  |  |  |  |
| **_Fe_** | _.652**_ | _-0.288_ | _0.067_ | _-0.173_ | _.611**_ | **_.868**_** | _0.074_ | _-0.242_ | _0.315_ | _-0.004_ | _1_ |  |  |  |  |  |  |  |  |  |  |  |  |  |  |
| **_Co_** | **_.721**_** | _-0.298_ | _0.017_ | _-0.238_ | _.564**_ | **_.891**_** | _0.032_ | _-0.272_ | _.406*_ | _0.024_ | **_.912**_** | _1_ |  |  |  |  |  |  |  |  |  |  |  |  |  |
| **_Ni_** | _0.117_ | _0.199_ | _.427*_ | _.444*_ | _0.221_ | _0.112_ | _.526**_ | _0.117_ | _0.163_ | _0.178_ | _0.107_ | _0.051_ | _1_ |  |  |  |  |  |  |  |  |  |  |  |  |
| **_Cu_** | _0.302_ | _.660**_ | **_.781**_** | _.748**_ | **_.718**_** | _0.261_ | **_.898**_** | _.593**_ | **_.709**_** | _.650**_ | _0.304_ | _0.262_ | _.454**_ | _1_ |  |  |  |  |  |  |  |  |  |  |  |
| **_Zn_** | _0.33_ | _0.289_ | _.564**_ | _.352*_ | _.528**_ | _.351*_ | _.523**_ | _0.284_ | _.405*_ | _.631**_ | _.375*_ | _0.284_ | _0.256_ | **_.710**_** | _1_ |  |  |  |  |  |  |  |  |  |  |
| **_Ga_** | _0.018_ | _.623**_ | _.711**_ | _.503**_ | _.555**_ | _0.092_ | _.602**_ | _.578**_ | _.485**_ | _.402*_ | _0.163_ | _0.01_ | _.417*_ | **_.733**_** | _.676**_ | _1_ |  |  |  |  |  |  |  |  |  |
| **_Se_** | _0.247_ | _-0.085_ | _-0.048_ | _-0.071_ | _-0.116_ | _0_ | _-0.062_ | _-0.1_ | _-0.02_ | _.591**_ | _-0.123_ | _-0.035_ | _-0.074_ | _-0.011_ | _0.329_ | _-0.234_ | _1_ |  |  |  |  |  |  |  |  |
| **_Sr_** | _-0.135_ | _0.091_ | _0.196_ | _0_ | _-0.153_ | _-0.134_ | _0.081_ | _0.028_ | _0.107_ | _0.187_ | _-0.184_ | _-0.075_ | _-0.126_ | _0.053_ | _0.168_ | _-0.041_ | _.490**_ | _1_ |  |  |  |  |  |  |  |
| **_Ag_** | _-0.104_ | **_.942**_** | _.615**_ | _.645**_ | _.429*_ | _-0.29_ | _.534**_ | **_.978**_** | _.586**_ | _.668**_ | _-0.288_ | _-0.316_ | _0.099_ | _.583**_ | _0.297_ | _.581**_ | _-0.06_ | _0.029_ | _1_ |  |  |  |  |  |  |
| **_Cd_** | _0.295_ | _-0.063_ | _-0.035_ | _-0.05_ | _-0.014_ | _0.049_ | _-0.056_ | _-0.051_ | _0.019_ | _.596**_ | _-0.067_ | _-0.01_ | _-0.057_ | _0.002_ | _0.336_ | _-0.226_ | **_.829**_** | _0.084_ | _0.009_ | _1_ |  |  |  |  |  |
| **_In_** | _0.262_ | _0.083_ | _0.181_ | _0.171_ | _0.064_ | _0.031_ | _0.231_ | _0.041_ | _0.09_ | **_.695**_** | _-0.039_ | _-0.006_ | _0.168_ | _0.256_ | _.527**_ | _0.009_ | **_.822**_** | _0.172_ | _0.097_ | _.910**_ | _1_ |  |  |  |  |
| **_Ba_** | _0.24_ | _-0.076_ | _-0.054_ | _-0.065_ | _-0.115_ | _-0.018_ | _-0.069_ | _-0.083_ | _-0.021_ | _.606**_ | _-0.141_ | _-0.056_ | _-0.085_ | _-0.023_ | _0.327_ | _-0.248_ | **_.987**_** | _.406*_ | _-0.035_ | _.901**_ | **_.877**_** | _1_ |  |  |  |
| **_Hg_** | _0.013_ | _.535**_ | _0.203_ | _0.061_ | _0.136_ | _-0.178_ | _-0.021_ | _.501**_ | _0.301_ | _.505**_ | _-0.252_ | _-0.209_ | _-0.096_ | _0.144_ | _0.089_ | _0.237_ | _0.231_ | _0.141_ | _.544**_ | _0.111_ | _0.069_ | _0.21_ | _1_ |  |  |
| **_Pb_** | _-0.033_ | _.353*_ | _.496**_ | _.437*_ | _0.033_ | _-0.17_ | _.489**_ | _0.218_ | _0.14_ | _.488**_ | _-0.169_ | _-0.215_ | **_.743**_** | _.466**_ | _.420*_ | _.473**_ | _0.332_ | _0.052_ | _0.247_ | _0.246_ | _.495**_ | _0.314_ | _0.21_ | _1_ |  |
| **_Bi_** | _0.24_ | _-0.065_ | _0.048_ | _-0.058_ | _0.01_ | _0.08_ | _-0.034_ | _-0.034_ | _0.05_ | _.520**_ | _-0.044_ | _0.023_ | _-0.07_ | _0.003_ | _.416*_ | _-0.145_ | **_.766**_** | _0.27_ | _0.022_ | **_.912**_** | **_.858**_** | **_.840**_** | _0.052_ | _0.149_ | _1_ |

*. Correlation is significant at the 0.05 level (2-tailed). **. Correlation is significant at the 0.01 level (2-tailed).


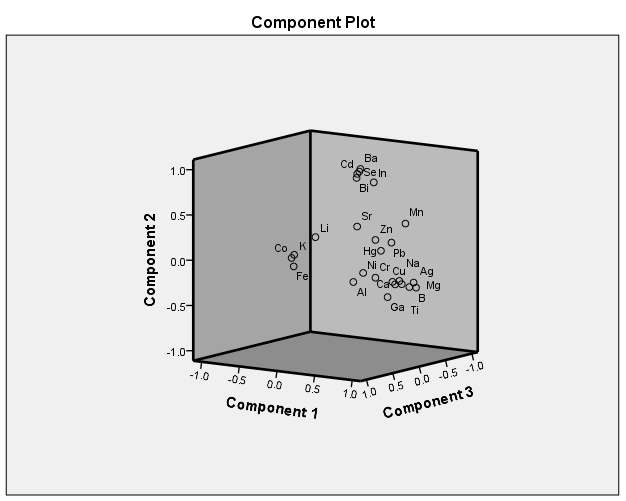


**Figure S1**. Principal component analysis (PCA) of the 25 elements detected in the sediments at the studied sites.

Table S11. The Principal component analysis of the 25 elements detected in the sediments at the studied locations.

| Component Matrix^a^ | | | |
| --- | --- | --- | --- |
| Metals | Component | | |
|  | 1 | 2 | 3 |
| Li | 0.266 | 0.344 | 0.761 |
| B | 0.801 | -0.326 | -0.384 |
| Na | 0.804 | -0.205 | -0.062 |
| Mg | 0.732 | -0.270 | -0.211 |
| Al | 0.669 | -0.132 | 0.617 |
| K | 0.119 | 0.161 | 0.953 |
| Ca | 0.819 | -0.226 | 0.039 |
| Ti | 0.757 | -0.315 | -0.320 |
| Cr | 0.731 | -0.126 | 0.287 |
| Mn | 0.848 | 0.426 | -0.116 |
| Fe | 0.101 | 0.029 | 0.936 |
| Co | 0.069 | 0.119 | 0.932 |
| Ni | 0.434 | -0.131 | 0.095 |
| Cu | 0.914 | -0.163 | 0.224 |
| Zn | 0.713 | 0.286 | 0.262 |
| Ga | 0.739 | -0.371 | 0.068 |
| Se | 0.184 | 0.923 | -0.188 |
| Sr | 0.120 | 0.301 | -0.239 |
| Ag | 0.770 | -0.272 | -0.381 |
| Cd | 0.210 | 0.910 | -0.112 |
| In | 0.423 | 0.837 | -0.121 |
| Ba | 0.189 | 0.950 | -0.204 |
| Hg | 0.369 | 0.045 | -0.335 |
| Pb | 0.562 | 0.166 | -0.258 |
| Bi | 0.220 | 0.870 | -0.087 |
| Total | 8.39 | 5.244 | 4.632 |
| % of variance | 33.398 | 20.974 | 18.529 |
| % of cumulative | 33.398 | 54.372 | 72.901 |

Extraction Method: Principal Component Analysis. a. 3 components extracted.

Table S12. Concentrations of some PTEs (μg/g dw) in the sediments of the present study compared to other parts along the Mediterranean coast.

| **Location** | **Cd** | **Cu** | **Mn** | **Pb** | **Zn** | **References** |
| --- | --- | --- | --- | --- | --- | --- |
| Alexandria Western Harbor - Bardaweel Lagoon, Egypt | 0.136 - 79.701 | 0.286 - 2.225 | 0.075 - 0.535 | 5.137 - 58.449 | 2.005 - 12.457 | This study |
| Manzala Lagoon, Egypt | 0.17-115.4 | 7.89-380 | 419.6-1550 | 9.6-146.8 | 48.8-12.15 | [2] |
| Edku Lagoon, Egypt | 1.47-7.3 | 18-60 | 115-1749 | 4-37.14 | 40-352 | [22 |
| Rosetta coast, Egypt | 21-37 | 7.6–42.7 | 450–640 | 214–476 | 53–388 | [3] |
| Between Damietta and Port Said, Egypt | -- | 1.62-14.69 | 47.58-257.1 | 1.16-19.07 | 7.99-400.3 | [4] |
| Mediterranean coast, Egypt | 0.4-0.47 | 0.46-26.2 | 17-1086 | 3.34-53.67 | 2.02-62.21 | [5] |
| Abu-Qir Bay, Egypt | 0.31-4.89 | 10.2-22.8 | 115-479.6 | 1.9-16.79 | 25.23-104 | [6] |
| Mediterranean coast, Libya | 5–10.5 | 9.1–22.7 | 14.3–49.4 | 8.9–56.9 | 11.6–30.5 | [7] |
| Mediterranean coast, Morocco | 0.14–0.27 | 4.09–29.1 | 256.5–651.6 | 33.1–47.9 | 64.82–110.77 | [8] |
| Safax coast, Tunisia | 0.2-2.5 | 40-415 | 552-2826 | 37-314 | 33-117 | [9] |
| Mediterranean coast, Egypt | 0.06-0.42 | 6.4-18.5 | 107-1090 | 5.3-57 | 12.3-59 | [10] |

**References**

1. USDOE, 2011. The Risk Assessment Information System (RAIS). U.S. Department of Energy’s Oak Ridge Operations Office (ORO).
2. Shalaby, B., Samy, Y. M., Mashaly, A. O., El Hefnawy, M. A. A. Comparative Geochemical Assessment of Heavy Metal Pollutants among the Mediterranean Deltaic Lakes Sediments (Edku, Burullus and Manzala), *Egypt. Egyptian Journal of Chemistry*, **60(3),** 361–378 (2017).
3. El-Sorogy, A. S., Tawfik, M., Almadani, S. A., Attiah, A. Assessment of toxic metals in coastal sediments of the Rosetta area, Mediterranean Sea, Egypt. *Environmental Earth Sciences*, 75, 1–11 (2016).
4. El-Hamid, H. T. A., Hegazy, T. A., Ibrahim, M. S., El-Moselhy, K. M. Assessment of heavy metals pollution in marine sediments along the Mediterranean Sea, Egypt. J*ournal of Geography, Environment and Earth Science International,* **7(4)**, 1–11 (2016).
5. Soliman, N. F., Nasr, S. M., Okbah, M. A. Potential ecological risk of heavy metals in sediments from the Mediterranean coast, Egypt. J*ournal of Environmental Health Science and Engineering*, **13**, 1–12 (2015).
6. Ghani, S. A., El Zokm, G., Shobier, A., Othman, T., Shreadah, M. Metal pollution in surface sediments of Abu-Qir bay and Eastern harbour of Alexandria, Egypt. The *Egyptian Journal of Aquatic Research*, **39(1)**, 1–12 (2013).
7. Nasr, S. M., Okbah, M. A., El Haddad, H. S., Soliman, N. F. Fractionation profile and mobility pattern of metals in sediments from the Mediterranean Coast, Libya. *Environmental Monitoring and Assessment*, **187**, 1–8 (2015).
8. Omar, M. *et al*. Distribution of heavy metals in marine sediments of Tetouan coast (North of Morocco): natural and anthropogenic sources. *Environmental Earth Sciences*, **74**, 4171–4185 (2015).
9. Díaz-de Alba, M., Galindo-Riano, M. D., Casanueva-Marenco, M. J., García-Vargas, M., & Kosore, C. M. Assessment of the metal pollution, potential toxicity and speciation of sediment from Algeciras Bay (South of Spain) using chemometric tools. *Journal of Hazardous Materials*, **190(1–3)**, 177–187 (2011).
10. El Baz, S.M. and Khalil, M.M. Assessment of trace metals contamination in the coastal sediments of the Egyptian Mediterranean coast. *Journal of African Earth Sciences,* **143**, 195-200 (2018).
